# Sulforaphane and Its Bifunctional Analogs: Synthesis and Biological Activity

**DOI:** 10.3390/molecules27051750

**Published:** 2022-03-07

**Authors:** Łukasz Janczewski

**Affiliations:** Faculty of Chemistry, Institute of Organic Chemistry, Lodz University of Technology, Zeromskiego 116, 90-924 Lodz, Poland; lukasz.janczewski@p.lodz.pl

**Keywords:** sulforaphane, isothiocyanates, anticancer activity, antibacterial activity, analogs of sulforaphane

## Abstract

For decades, various plants have been studied as sources of biologically active compounds. Compounds with anticancer and antimicrobial properties are the most frequently desired. Cruciferous plants, including Brussels sprouts, broccoli, and wasabi, have a special role in the research studies. Studies have shown that consumption of these plants reduce the risk of lung, breast, and prostate cancers. The high chemopreventive and anticancer potential of cruciferous plants results from the presence of a large amount of glucosinolates, which, under the influence of myrosinase, undergo an enzymatic transformation to biologically active isothiocyanates (ITCs). Natural isothiocyanates, such as benzyl isothiocyanate, phenethyl isothiocyanate, or the best-tested sulforaphane, possess anticancer activity at all stages of the carcinogenesis process, show antibacterial activity, and are used in organic synthesis. Methods of synthesis of sulforaphane, as well as its natural or synthetic bifunctional analogues with sulfinyl, sulfanyl, sulfonyl, phosphonate, phosphinate, phosphine oxide, carbonyl, ester, carboxamide, ether, or additional isothiocyanate functional groups, and with the unbranched alkyl chain containing 2–6 carbon atoms, are discussed in this review. The biological activity of these compounds are also reported. In the first section, glucosinolates, isothiocyanates, and mercapturic acids (their metabolites) are briefly characterized. Additionally, the most studied anticancer and antibacterial mechanisms of ITC actions are discussed.

## 1. Introduction

### 1.1. Isothiocyanates—General Properties

#### 1.1.1. Glucosinolates

**Figure 1:**
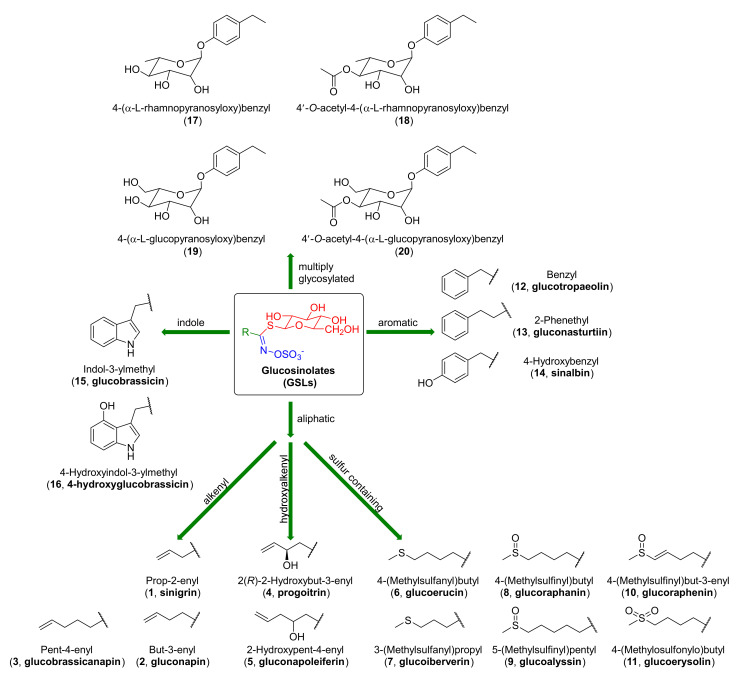
Structures of the main groups of glucosinolates.

The Brassicaceae family [1] also called Cruciferae, includes more than 2000 plant species. Among them, many are edible plants, such as Brussels sprouts, broccoli, radish, horse radish, cabbage, wasabi, and rocket [2,3]. These vegetables are characterized by high chemopreventive activity. International research [4,5] shows that their consumption decreases the risk of lung [6], breast [7], colon [8], and prostate cancers [9]. The high chemopreventive effects of Cruciferae, compared to other plants, are associated with the high content of glucosinolates (GSLs). GSLs [10,11,12,13,14,15,16,17] were discovered more than 200 years ago and they contain sulfur secondary metabolites. In 1956, Ettlinger and Lundeen [18] proposed general structures of GSLs (Figure 1). GSLs are composed of three elements: β-D-thioglucose group (in red), sulfonated oxime moiety (in blue), and side chain R (in green), whose structures correspond to the α-amino acids used during biosynthesis (Figure 1). The structures of GSLs were confirmed by synthesis in 1957 [19]. To date, more than 200 GSLs have been identified, and due of the structure of side chain R, they are divided into three main groups: aliphatic (**1**–**11**), aromatic (**12**–**14**), and indole derivatives (**15**–**16**). Aliphatic GSLs covering more than 50% of glucosinolates are divided into: alkenyl (**1**–**3**), hydroxyalkenyl (**4**–**5**), and those containing sulfur on II (**6**–**7**), IV (**8**–**10**), and VI (**11**) oxidation states [20]. In addition, GSLs can be found in other plants—different from Brassicaceae—such as Moringaceae [21,22,23], of which, the most widely cultivated is *Moringa oleifera*. GSLs with glycosylated R-groups belong to this group (**17**–**20**) (Figure 1).

**Figure 2:**
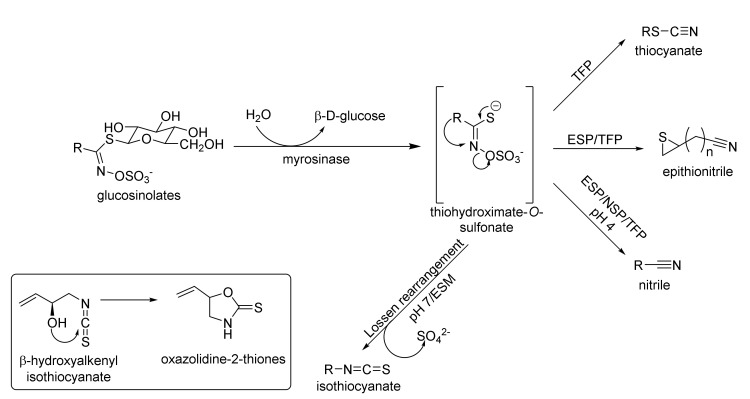
Enzymatic degradation of glucosinolates.

In plants, GSLs are stored in vacuoles, which protect them against degradation caused by myrosinase (β-D-thioglucosidase) (EC 3.2.1.1) [24,25]. In plants, the glucosinolate–isothiocyanate system has defense functions against insects, pathogens, and herbivores [26]. Cell damage, e.g., while chewing the plants, results in the formation of a variety of glucosinolate breakdown products (Figure 2). In mammals, GSLs are converted to isothiocyanates, through the bacteria present in the digestive tract. Reducing the amount of bacterial flora, as a result of using antibiotics, eliminates this pathway [27].

The breakdown of GSLs initiated by enzymatic hydrolysis of the thioglucoside bond leads to the elimination of β-D-glucose and the formation of an unstable thiohydroximate-*O*-sulfonate, which, depending on the pH, undergoes the so-called Lossen rearrangement to isothiocyanate (pH 7), or a decomposition to nitrile (pH 4). It was shown that a low pH inhibits the Lossen rearrangement [28]. For unstable β-hydroxyalkenyl isothiocyanates, subsequent cyclization to oxazolidine-2-thione takes place (Figure 2). Under certain conditions, plants modify the direction of aglucon degradation in the presence of specific proteins, such as the epithiospecifier protein (ESP) [29], the nitrile-specifier protein (NSP) [30], and the thiocyanate-forming protein (TFP) [31], catalyzing formation of epithionitrile, nitrile, or thiocyanate, respectively. On the other hand, the epithiospecifier modifier protein (ESM) inhibits the formation of nitrile and favors the formation of isothiocyanates [32] (Figure 2). Detailed information about enzymatic hydrolysis of glucosinolates and the functions of specifier proteins were reviewed by Burow and Wittstock [33].

#### 1.1.2. Isothiocyanates

**Figure 3:**
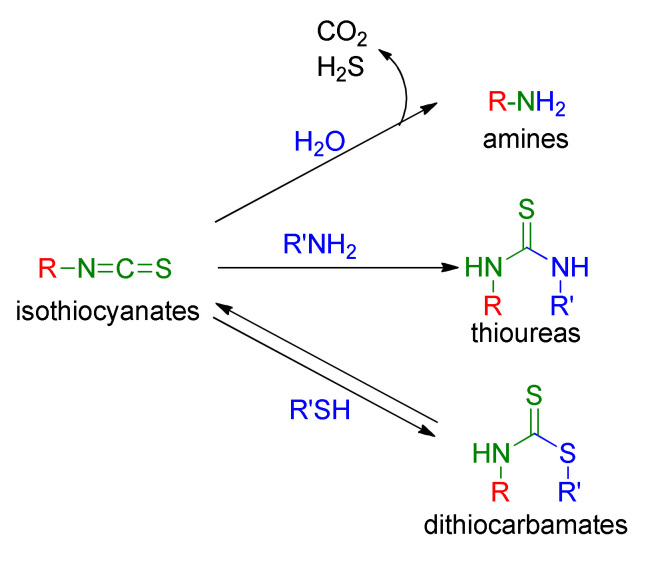
Reactions of isothiocyanates.

Biologically inactive GSLs undergo enzymatic transformations to biologically active isothiocyanates (ITCs). ITCs [34,35,36,37] are low molecular weight heterocummulenes with a general formula R–N=C=S (R-NCS), where R may be a structurally diverse aliphatic, aromatic or heterocyclic substituent. They are characterized by sharp and pungency odors [38] and relatively high volatility. Due to the presence of a reactive electrophilic carbon atom in the isothiocyanate group (–NCS), these compounds easily react in physiological conditions, in a reversible way with thiols, resulting in sensitivity to pH dithiocarbamates or forming thioureas in an irreversible reaction with amines. It was shown that ITCs react 1000 times faster with thiols than with amines [39]. Hydrolysis of ITCs leads to amines (Figure 3) [40].

**Figure 4:**
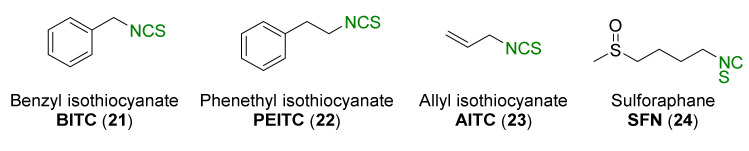
Structures of selected natural isothiocyanates.

Natural isothiocyanates, such as benzyl isothiocyanate (**21**, BITC) [41], phenethyl isothiocyanate (**22**, PEITC) [42,43,44], allyl isothiocyanate (**23**, AITC) [45], and the best known and the studied sulforaphane (**24**, SFN) [46,47,48] (Figure 4) have shown chemopreventive properties.

ITCs inhibit the carcinogenesis process and tumor development in vitro and in vivo by inhibiting the activity of carcinogens, inhibiting the cell cycle, activating apoptosis, and inhibiting metastasis. These compounds (due to their reactivities) also modify many proteins involved in cancer processes [49,50]. They inhibit cytochrome P450 enzymes, activate phase II enzymes by activating the Nrf2 factor, affect cell cycle regulators and Bcl-2 proteins, activate caspases, and inhibit Nf-κB factor [51,52]. The anticancer mechanisms of isothiocyanates, including SFN, are described in detail in Section 2.1. The large number of proteins potentially reacting with ITCs shows that ITCs do not have one particular molecular target. This is the advantage of ITCs, because it makes it more difficult for cancer cells to become resistant to ITCs. On the other hand, this feature can be a disadvantage, as it makes studying the anticancer mechanisms of isothiocyanate much more difficult. In addition to chemopreventive properties, ITCs have antibacterial properties [53,54] (described in detail in Section 2.2). They are also used as herbicides and fungicides [55]. Isothiocyanates also play an important role in organic synthesis as substrates in the synthesis of heterocyclic compounds [56,57], thioamides [58], and thiourea organocatalysts [59]. ITCs are also exploited as molecular probes [60,61].

SFN–1-isothiocyanato-4-(methylsulfinyl)butane (**24**) was, for the first time, “obtained” by Schmid and Karrer in 1948 [62]. It is the best known (and studied) isothiocyanate. It was, for the first time, isolated by Zhang [63] in 1992, from broccoli, where its concentration ranged between 0.8 and 21.7 μmol/g d wt [64]. Isolation of SFN led to increased interest in this compound, as well as other isothiocyanates, as confirmed by a large number of scientific papers on this subject. Analysis of the Web of Science database shows that, since 1992, about 3890 articles have been published on SFN, and over 5600 on isothiocyanates [65].

SFN, due to the presence of the chiral center on the sulfur atom, occurs as two enantiomers—natural (*R*)-SFN and synthetic (*S*)-SFN. Most tests used racemic SFN; however, studies confirm that (*R*)-SFN has biological activity [66]. The name of the “most popular isothiocyanate”, SFN, is due to its ability to simultaneously modify many cellular targets associated with cancer development, including DNA protection, by inhibiting the activity of mutagenic factors (phase I) and activation of phase II factors responsible for detoxification, inhibiting the proliferation of cancer cells and activating apoptosis, thereby limiting the process of multiplication of mutated cancer cells, and inhibiting the process of neogenesis and metastasis. SFN is able to prevent, remove, and reverse preneoplastic lesions [67]. Recent research by Chlopicki et al. [68] shows that L-SFN also exhibits antioxidant and protective effects on endothelial cells. All of these features mean that SFN, according to the US National Cancer Institute, is one of the 40 most promising anticancer compounds [69].

**Figure 5:**
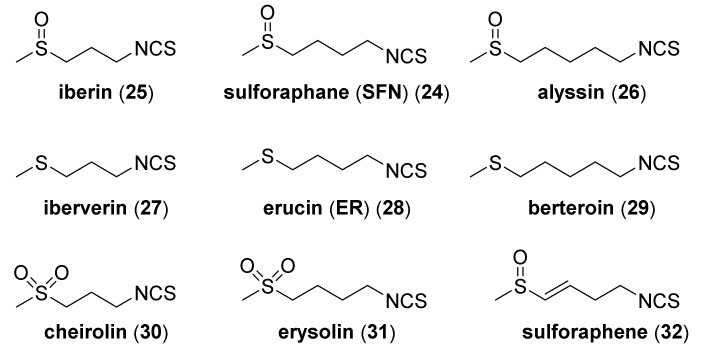
Structures of natural analogs of SFN.

When writing about SFN—its natural analogues, possessing a sulfur atom on II, IV, and VI oxidation states, and an alkyl chain containing 3 to 5 carbon atoms, should also be mentioned (Figure 5).

These include: iberin (**25**) and alyssin (**26**) [70], with methylsulfinyl group, iberverin (**27**) [71], erucin (ER) (**28**) [72] and berteroin (**29**) [73] with the methylsulfanyl group, and cheirolin (**30**) [74], and erysolin (**31**) [75] with the methylsulfonyl group. The α,β-unsaturated analog of sulforaphene (**32**) is also known [76] (Figure 5).

#### 1.1.3. Mercapturic Aid

**Figure 6:**
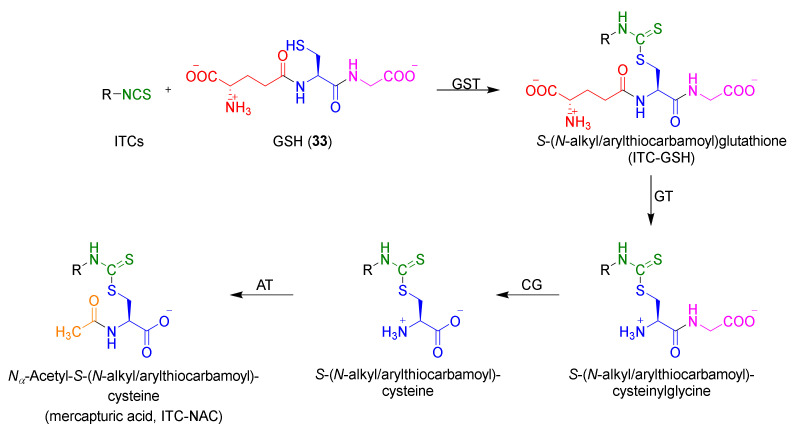
Metabolism of ITCs via the mercapturic acid pathway. R, an aliphatic or aromatic substituent.

After entering ITCs into the cell, glutathione (GSH, **33**) [77,78,79,80,81,82] is the first target of ITCs. In the cell, under the influence of glutathione *S*-transferase (GST), an immediate reaction of ITCs occurs with the -SH group of the cysteine residue of GSH. The *S*-(*N*-alkyl/arylthiocarbamoyl)glutathione (ITC–GSH) is formed, initiating the process of isothiocyanate metabolism, called the mercapturic acid pathway. The ITC–GSH under the influence of γ-glutamyl transferase (GT) dipeptidase (cysteinoglycinase (CG)) and *N*-acetyltransferase (AT) (transformation to conjugate with cysteinylglycine, cysteine, and *N*-acetylcysteine), giving mercapturic acids (ITC–NACs) as the final products of intracellular metabolism of ITCs (Figure 6) [40].

Compared to conjugates with cysteinylglycine and cysteine, which are formed in the intercellular space, mercapturic acid is formed in the liver, is transported to the kidneys, and removed with urine [40]. Studies have shown that in the urine of people who consume cruciferous plants, mercapturic acid is the main metabolite [83]. It was found that after 8 h of ingestion of broccoli sprouts, about 60% of the ITCs are removed in the urine [84]. Detailed studies show that 7% is pure SFN, less than 1% an SFN–GSH conjugate and SFN conjugate with cysteinylglycine, about 28% an SFN conjugate with cysteine, and about 65% ITC–NAC formed from SFN [85,86].

**Figure 7:**
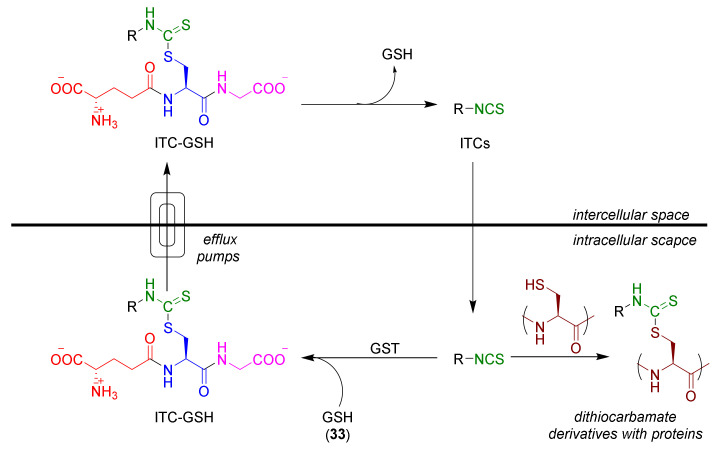
Accumulation of ITCs in the cell.

The pharmacokinetics of ITCs [87] lead to intracellular accumulation (Figure 7). 

After diffusing into the cell, ITCs in the presence of GST undergo rapid reactions with GSH (**33**), leading the formation of the ITC–GSH conjugate. This compound, under the influence of efflux pumps, moves to the intercellular space, where it breaks down in the hydrolysis reaction with recovery of the original ITCs, which again enter the cell. The result of this process is the depletion of intracellular GSH and high accumulation of the ITCs in the cell (ITC concentration in the cell, compared to the concentration in the intercellular area, is 100–200 times larger) [88]. Glutathione depletion in the cell allows ITCs to react with other proteins containing cysteine residues (the presence of a thiol group). The consequences of this process are the increase of ROS in the cell, leading to the induction of various biological responses, including short-term, or a complete arrest of the cell proliferation process, as well as the activation of apoptosis or necrosis [89,90]. The formation via the mercapturic acid pathway leads to other biological active conjugates causing, e.g., inhibition of histone deacetylase activity [91], and assures the possibility of ITC reaction with other intercellular and intracellular proteins.

## 2. Mechanism Determining Biological Activity of ITCs

### 2.1. The Mechanism of Anticancer Activity

There are many excellent reviews on anticancer activities, as well as the mechanisms of action of ITCs [92,93,94,95,96] and SFN [97,98,99,100]. In vitro and in vivo tested ITCs, including SFN, show their biological activities at all stages of carcinogenesis. Generally, carcinogenesis consists of three stages: (i) initiation—a rapid and irreversible process leading to genotypic changes and DNA damage due to interaction with the carcinogen; (ii) promotion—the selective proliferation of initiated cells leading to the formation of pre-cancerous lesions; (iii) progression—transformation of benign pre-cancerous lesions into cancer. This section briefly summarizes the most studied ITC anticancer mechanisms.

#### 2.1.1. Initiation Stage

At the initiation stage, isothiocyanates exhibit chemopreventive activities by regulating xenobiotics metabolism. In the conversion of carcinogens and their elimination, two groups of enzymes participate: phase I enzymes and phase II enzymes.

##### Inhibition Phase I Enzymes and Activation Phase II Enzymes

Phase I enzymes belonging to the cytochrome P-450 (CYP) family catalyze oxidation, reduction, and hydrolysis reactions, preparing the carcinogen for further changes, which leads to metabolic activation of some procancerogens and the formation of metabolites capable of interacting with DNA and causing mutations [101]. ITCs inhibit and reduce the activity of CYP 1A1, 1A2, 2A6, 3A4, 2B1, 2D6, and 2E1 in cancer tissues [52]. Yokoi et al. [102] confirmed that PEITC (**22**) completely inhibits CYP 1A2, 2A6, 2B6, 2C9, 2C19, 2D6, 2E1, and 3A4 activity.

In phase II metabolism of carcinogens, a particular role is played by glutathione S-transferase (GST), NAD(P)H: quinone oxidoreductase (NQO1), UDP-glucuronyltransferase (UGT), quinone reductase (QR), and nicotinamide *N*-methyltransferase [103,104], enzymes responsible for increase the solubility of xenobiotics in water and facilitate their excretion [105]. In vitro studies on the HepG2 human hepatocyte line showed that the use of SFN (**24**) caused an increase in the concentration of mRNA UGT 1A1 and GST A1 [106], and an increase in the activity of NQO1 [107], accompanied by bilirubin glucuronidation [108].

One of the proposed mechanisms of stimulation of phase II enzymes by SFN concerns the activation of the Nrf2 [109] allowing the interaction with the antioxidant response element (ARE) encoding the detoxification enzyme genes. Under normal conditions, Nrf2 occurs in the cellular cytosol, and it is binding to the Kelch-like ECH-associated protein 1 (Keap1) leading the Nrf2–Keap1 complex. SFN, or other ITCs after entering the cell, react with thiol groups present on the surface of the Keap1, resulting in the degradation of the Nrf2–Keap1 complex. The released and activated Nrf2 migrates to the cell nucleus, where it binds to ARE and stimulates transcription of genes encoding phase II enzymes [109]. Studies have shown that the activation of Nrf2, apart from what is described above, can also take place due to the activation of the MAPK protein kinase pathway [46].

#### 2.1.2. Promotion Stage

At the promotion stage, SFN (and other ITCs) show antiproliferative activity against tumor cells by, e.g., inhibiting the cell cycle, inducing apoptosis or inhibiting histone deacetylase (HDAC).

##### Inhibition of the Cell Cycle

Tumor cells are characterized by rapid growth resulting from the loss or disturbed functioning of mechanisms of cell cycle regulation. Cyclin-dependent kinases (CDKs), cyclins, and cyclin-dependent kinase inhibitors are involved in controlling the cycle of healthy cells. The formation of a cyclin and CDK complexes permit the cell to pass through the next phases of the cycle, while the CDK inhibitors stop the cycle in a specific phase. The research has shown that SFN acts as a cell cycle regulator, inhibiting the cell cycle in the G2/M phase [110]. Crossing the G2/M point requires activation of a complex of the cyclin B and CDK1. Phosphorylation of CDK1 by Wee1 and Myt1 kinases leads to inactivation of the complex, while the activity of the Cdc25 protein phosphatase conditions the activity of the complex, allowing to go to the M phase. The Cdc25 protein phosphatase is regulated by Chk1 and Chk2 kinases, and phosphorylation of Cdc25 inactivates it and leads to the deactivation of the cyclin B and CDK1 complex, finally stopping the cell cycle in the G2/M phase [111]. Studies have shown that SFN prevents the transfer of PC-3 cancer cells to the M phase of the cell cycle by decreasing the levels of cyclin B1, Cdk25B, and Cdk25C, and increasing phosphorylation of Cdk25C by Chk2 [112]. In addition, it was shown that one of the mechanisms of action of SFN on the HT-29 colorectal tumor cell line is the expression of protein p21 [113]. Studies on the same cell line have shown that ITCs also inhibits the cell cycle in the G1 phase by reducing regulation of cyclin A, D, and E [114]. The apoptotic effect depends mainly on the dose of the compound and the time of exposure to it [115]. Studies on the Caco-2 cell line have shown that SFN at a concentration of 20 μM inhibits the cell cycle in the G2/M phase, while at a concentration above 20 μM, it inhibits the G1 phase. In addition, the short-term exposure of tumor cells to SFN resulted in reversible inhibition of the cell cycle in the G2/M phase, while complete inhibition of the cell cycle required more than 12 h of incubation [116].

##### Inducing Apoptosis

Apoptosis, programmed cell death, is one of the most commonly used strategies to fight cancer cells. In some cases, the low susceptibility of cancer cells to signals triggering the process of apoptosis contributes to the development of tumors. SFN is involved in activating signals that lead to apoptosis in many types of cancer cells. SFN stimulates the formation of apoptotic bodies, reduces the concentration of anti-apoptotic Bcl-2 and Bcl-XL proteins, increases the expression of the proapoptotic Bax protein, the activation of caspase 3, and the degradation of the poly(ADP-ribose) polymerase, and thus interacts on the mitochondrial-dependent apoptosis factors [117]. In addition, SFN is responsible for decreasing the activity of apoptosis inhibitors (IAP: cIAP1, cIAP2, and XIAP) and the induction of the Apaf-1 protein [118]. In addition to SFN, other natural isothiocyanates participate in the process of apoptosis. BITC activates procaspases-8 and -9 [119]. AITC and PEITC increase the level of the t-Bid proapoptotic protein in HL-60 leukemia cells [120], and AITC further reduces the Bcl-XL protein concentration in LNCaP prostate cancer cells [121]. Mechanistic studies suggest that the generation of the reactive oxygen species (ROS) by SFN induces the mechanism of cancer cell death. Tests performed on the human prostate tumor cell PC-3 line showed that ROS generated in the presence of SFN changed the potential of the mitochondrial membrane, leading to the release of cytochrome C from the intermembrane space to the cytoplasm [122]. Cytochrome C, together with the Apaf-1 protein and ATP, form an apoptosome, activating procaspase-9. The apoptosome and caspase-9 complex recruits and activates procaspase-3 and/or procaspase-7, leading to apoptosis [123].

##### Inhibiting Histone Deacetylase (HDAC)

Histones undergo reversible acetylation of selected *N*-terminus lysine residues. The modification occurring in the presence of acetyltransferase leads to serious changes at all levels of chromatin structures, causing disorders of the chromatin, folding into higher-order structures [124], increasing its solubility under physiological conditions and, most importantly, favor transcription [125]. Unlike acetylation, deacetylation of histones leads to the blockage of chromatin structures and, as a consequence, to the termination of the transcription process. Histone deacetylase is associated with many cancers, it causes suppression of transcription and affects the dysregulation of mechanisms controlling the cell cycle and apoptosis. In addition, HDAC by deacetylation of tumor suppressor genes, e.g., the *p21* gene, leads to silencing of their transcriptions or their complete deactivation. In vitro studies performed on the HCT 116 cell line showed that SFN at concentrations of 3–15 μM causes a decrease in HDAC activity [126]. The process of inhibiting histone deacetylase is combined with the inhibition of the cell cycle and induction of apoptosis. Studies show that inhibition of HDAC with SFN contribute to inhibition of the cell cycle in PC-3 cells in the G2/M phase [127], while inhibition of HDAC using BITC causes the deactivation of NF-κB, which leads to decreased activity of cyclin D1 and, consequently, to the inhibition of the cell cycle [128]. In addition, as a consequence of the inhibition of histone deacetylase with SFN, researchers observed an increase in the concentration of p21 and Bax proteins [126], which are involved in the process of apoptosis.

#### 2.1.3. Progression Stage

At the stage of progression, isothiocyanates, as well as SFN, are responsible for inhibiting the process of angiogenesis and metastasis.

##### Inhibition of Angiogenesis and Metastasis

Angiogenesis is the process of formation of new blood vessels [129]. It is claimed that it is an important stage in tumor growth and metastasis, as a result of which, oxygen and nutrients are supplied to the formed cancer. For this reason, the stage of angiogenesis has become the target of anticancer therapies. The main cytokinin initiating the process of angiogenesis is the vascular endothelial growth factor (VEGF), responsible for an increase in vascular permeability and stimulating proteolytic enzymes. In addition to VEGF, fibroblast growth factor 2 (FGF-2) and epidermal growth factor (EGF) are important factors involved in angiogenesis [130]. 

Metastasis is the ability to spread cancer cells from the primary outbreak to lymph nodes, and to tissues and organs, and is a hallmark of malignant tumors. The metastasis process requires the activation of proteolytic enzymes, e.g., metalloproteinases (MMPs). These enzymes belong to the family of zinc-containing enzymes and are capable of degrading the basement membrane, which is necessary to penetrate endothelial cells into new places and create new vessels. MMPs are overexpressed in cancer cells [131]. Studies have shown that PEITC has an anti-angiogenic effect by inhibiting the activity of VEGF and EGF [132]. Additionally, tests on human umbilical vein endothelial cells (HUVECs) with SFN have demonstrated that this compound is involved in the regulation of the various stages of angiogenesis, by reducing vascular formation and propagation of endothelial cells [133]. In addition, SFN inhibits metalloproteinase-9 activity and reduces the metastatic ability of MDA-MB-231, a triple-negative breast cancer cell line.

### 2.2. Antibacterial Activity

Antibacterial properties of ITCs are not as wide tested as anticancer properties. To date, only two extensive reviews have described the antibacterial activities of ITCs. Dufour et al. [53] in 2015 described, in detail, the antibacterial modes of action, e.g., the effects on influencing the membrane, inhibition of enzymic or regulatory activities, the effect of ITCs on respiratory enzymes, the induction of heat shock and oxidative stress responses, and the induction of a stringent response of natural ITCs (SFN, BITC, PEITC, etc.), as well as mechanisms of resistance to ITCs. Moreover, Romeo et al. [54], in 2018, described the antibacterial properties of natural ITCs against Gram-positive (*H. pylori*, *S. aureus,* etc.) and Gram-negative (*P. aeruginosa*, *E. coli*, etc.) bacteria. For this reason, in this review, the antibacterial activities of ITCs are generally and briefly characterized.

In Japan, ITCs are used as natural food additives to protect them against microorganisms [134]. Highly volatile allyl isothiocyanate (AITC) plays a special role. It is used in antimicrobial food packaging to reduce, inhibit, and delay the growth of microorganisms in packed food. ITCs obtained from cruciferous vegetables, most often from horseradish, wasabi, or radish, are also added to food as spices [135]. The bacteriostatic and bactericidal effect of ITCs depend on the dose, and the concentration responsible for the above-mentioned effects is comparable or lower than the concentration of classical antibiotics used for the same purposes. ITCs show synergism with commonly used antibiotics. For example, a solution of p-hydroxyphenethyl isothiocyanate in glucose exhibits synergism with aminoglycoside antibiotics and streptomycin in relation to *E. coli* and *S. aureus* [136]. Moreover, AITC and phenethyl isothiocyanate (PEITC) show synergism with streptomycin against some Gram-negative bacteria (*E. coli* or *P. aeruginosa*) [137]. However, it was shown that even a small change in the concentration of isothiocyanate or antibiotic can cause the opposite effect and suppress antibacterial effect [138]. The mechanism of synergism, or its quenching caused by ITCs, is not yet known. Bacteriostatic properties of ITCs have also been noticed in agriculture, and ITCs are used to reduce the population of bacteria found in soil; for this purpose—genetically modified *Arabidopsis thaliana* (radish) plants using a transgenic *A*. *thaliana* that overexpress glucosinolate with p-hydroxybenzyl substituent (**14**, sinalbin, Figure 1) [139]. Glucosinolates, by diffusion, are transported from the roots to the rhizosphere, where under the action of extracellular myrosinase, they are transformed into ITCs [140].

#### Mechanism of Antibacterial Activity

The mechanism of antibacterial activity of ITCs is not as well understood. It is claimed that the antibacterial activity of ITCs is associated with disintegration of the cell membrane (causing the outflow of all metabolites), inhibition of bacterial quorum sensing—a system of communication between bacteria through autoinducers, inhibition of biofilm production, inhibition of enzymes necessary for the proper functioning of bacteria, induction of thermal shock, or induction of oxidative stress. The most commonly studied Gram-negative and Gram-positive bacteria strains and the antibacterial mechanisms of ITCs are briefly discussed in this section. 

Regarding *H. pylori* (Gram-negative) [141], SFN has the highest activity in relation to this strain, as well as to resistant strains of *H. pylori* (an inhibition of SFN is similar to the inhibition of antibiotics—clarithromycin and metronidazole) [142]. It is known that stomach infections by *H. pylori* are possible due to the ureases [143] converting urea into ammonia, resulting in the neutralization of acidic gastric juices [144]. SFN and other ITCs likely inhibit the activity of *H. pylori* urease, but this is still the subject of many studies [145]. 

Regarding Ca. *jejuni* (Gram-negative), benzyl isothiocyanate (BITC) is the most active natural isothiocyanate in relation to both antibiotic-resistant and -sensitive strains, Ca. *jejuni* [146]. Studies show that antibacterial activity of BITC is associated with the activation of metabolic pathways responsible for thermal shock and oxidative stress, leading to protein aggregation, energy metabolism disorders and, finally, bacterial death [147]. 

BITC also has the highest activity against *S. enterica* [148] (Gram-negative) and disintegrates cell membranes [149].

Enterohemorrhagic *E. coli* (EHEC) (Gram-negative) [150], whose strain O157:H7 produces the Shiga toxin, is the most studied, enteric, pathogenic *E. coli* strain. AITC is characterized by antibacterial activity on the *E. coli* O157:H7 strain, similar to polymyxin B, causing cell membrane disintegration and metabolite efflux and, consequently, bacterial death [151]. AITC inhibits two key enzymes in the metabolism of bacteria: thioredoxin reductase, involved in the synthesis of ribonucleotides, and acetate kinase, which is associated with energy metabolism [152]. From the group of natural ITCs (SFN, AITC, BITC, phenyl, and isopropyl isothiocyanate), the highest comparable to AITC activity in inhibiting *E. coli* EHEC, including *E. coli* O157:H7, showed BITC. In comparison to conventional antibiotics, the tested ITCs better inhibited the production of the Shiga toxin. Detailed studies have shown that the aforementioned ITCs affect penta/tetraphosphate (p)ppGpp, which influences RNA polymerase activity, bacteriophages development, and Shiga toxin production. ITCs increase the levels of penta/tetraphosphate (p)ppGpp, decrease the synthesis of RNA, and inhibit the development of prophageand the production of the Shiga toxin [153].

*P. aeruginosa* (Gram-negative) aerobic bacterium is able to colonize various environments and produce biofilm [154,155]. Many bacteria, including Pseudomonas strains, are equipped with a quorum sensing (QS) [156] system, by which bacteria communicate to coordinate gene expression. This allows them to control the expression of genes that are important for the entire population, by secreting and receiving signal molecules called autoinducers. This system controls biofilm formation, bioluminescence generation, the production of antibiotics and siderophores, and bacterial motility [157]. These coordinated behaviors allow bacteria to compete with multicellular organisms and survive sharp and sudden environmental changes. In *P. aeruginosa*, there are two main QS systems—*las* and *rhl*. The *las* system consists of synthesis *LasI*, the “autoinduction” gene responsible for the synthesis of autoinducers *N*-[3-oxo-dodecanoyl]-L-homoserine lactones (3-oxo-C12-HSL) and *lasR*, genes encoding transcription regulators. The *rhl* system consists of the pair *RhlI*/*RhlR*, which respond to *N*-butyryl homoserine lactones (C_4_-HSL) [158,159,160]. Studies on natural isothiocyanates (AITC, BITC, and PEITC) and the mixtures of those ITCs have shown that AITC and the “cocktail” of ITCs were characterized by the highest activity. However, only PEITC inhibited biofilm production [161]. In other studies, AITC and PEITC caused cell membrane disintegration and an increase in the hydrophilic nature of the membrane, changing its physicochemical properties [162]. Meijler et al. [163], with the *P. aeruginosa* strain, showed that by using SFN and its sulfide analog erucin (**28**, Figure 5), it is possible to affect bacterial QS. The consequence of this was the inhibition of the production of biofilm and pyocyanin—a cytotoxic dye that affects the central nervous system, urological system, and vascular system, causing inflammation [164]. Both biofilm and pyocyanin production are virulence factors controlled by QS. Iberin (**25**, Figure 5), an SFN analogue, also affects bacterial QS, by reducing the activity of the *RhlI*/*RhlR* expression [165]. 

Studies on the activities of natural ITCs (AITC, BITC, PEITC, and SFN) and mixtures of these ITCs (AITC, BITC, and PEITC) have shown that the mixtures show the highest activities in relation to the *S. aureus* [166,167] (Gram-positive) strain. Among individual ITCs, BITC had the best activity, while AITC was inactive. It was also shown that BITC, PEITC, and SFN, and their mixtures showed higher activity than vancomycin [168]. Contrary to earlier research suggesting that AITC is inactive against *S. aureus*, Lu et al. [169], in 2016, proved that AITC causes growth inhibition of *S. aureus,* and similar to PEITC, causes cell membrane disintegration and bacterial death [162].

### 2.3. Clinical Trials of SFN

In addition to in vitro and in vivo tests, SFN has also been selected for clinical trials; however, there is a limited number of these results in the literature. Talalay et al. [170] described a placebo-controlled, double-blind phase I clinical trial of healthy volunteers using extracts of sprouts containing either glucosinolates (principally glucoraphanin, the precursor of SFN) or ITCs (principally SFN). After 7 days of trials, no significant toxicities associated with taking the extracts at the doses employed were observed. In 2007, Visvanathan et al. [171] published research, where eight healthy women took an oral dose of broccoli sprout preparations containing 200 μmol of SFN. Studies demonstrated that sulforaphane distributed to the breast epithelial cells in vivo and exerted pharmacodynamic action in these target cells, consistent with its mechanism of chemoprotective efficacy. Fahey et al. [172] showed that administration of sulforaphane (100 μmol/day on 14 days) improved the bronchoprotection response in asthmatics who had an increase in NQO1 gene expression and did not have a decrease in their initial response to the MCh challenge. Therefore, SFN administration was able to improve a major defect of even mild asthma. Yanaka and co-workers [173] conducted research in which they fed forty-eight *H. pylori*-infected patients with broccoli sprouts (70 g/d; containing 420 μmol of SFN precursor) for 8 weeks. Results showed antibacterial effects of SFN on *H. pylori*, leading to reduced gastritis, as well as an indirect (systemic) effect by increasing the mammalian cytoprotective (phase II) response. More information about the clinical trials of SFN are in the review articles [174,175].

Although SFN was tested in phase I and phase II clinical trials, where it had good anticancer and antibacterial properties, it was never qualified as a drug. This may be due to the polymorphism of the genes for the GSTM1 and GSTT1 glutathione *S*-transferase isoenzymes. GST is involved in the detoxification of many chemical carcinogens and is responsible for the metabolism of ingested ITCs. Cohort studies indicate a protective effect of a diet rich in cruciferous vegetables against cancers of the lung, colon and breast only in patients with GSTM1 and GSTT1-null genotype [176]. Due to the fact that GST participates in the metabolism of SFN and thus affects its excretion, lower enzyme activity in people with GST gene polymorphism may result in slower elimination and longer exposure of cancer cells to this compound provided with the diet. In people with gene *GSTT1* a greater excretion of ITCs outside the body was observed, and thus a shorter time of exposure to cancer cells [177].

## 3. Methods of Synthesis of ITCs

**Figure 8:**
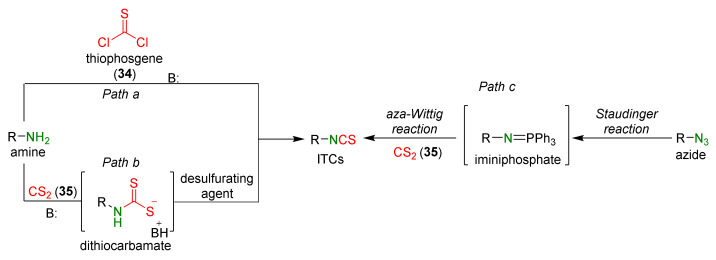
Synthesis of ITCs using amines (*Path a and b*) or azides (*Path c*) as substrates.

Many ITC synthesis methods are described in the literature. The choice of the synthetic method depends on the availability of the starting substrates as well as on the sensitivity of other functional groups present in the substrates to the reaction conditions. Methods on the synthesis of ITCs (known thus far) are described in the Houben-Weyl encyclopedia [178]; new aspects of the synthesis were described by Mukerjee and Ashare [56], Wentrup et al. [179], and Singh et al. [180]. Primary amines are used as starting materials in the synthesis of isothiocyanates (Figure 8).

The synthesis of ITCs from primary amines, or their salts, involve the use of thiocarbonyl reagents, such as thiophosgene (**34**) and its substitutes, or carbon disulfide (**35**). They “enable” either directly or indirectly via the intermediate dithiocarbamates transformation of amines into ITCs (Figure 8, *Path a* and *b*). Alternatively, azides may be applied as substrates. These in turn allow the preparation of ITCs via a tandem Staudinger/aza-Wittig reaction, through the intermediate iminophosphoranes (Figure 8, *Path c*).

### 3.1. Synthesis ITCs Using Thiophosgene and Its Substitutes

**Figure 9:**
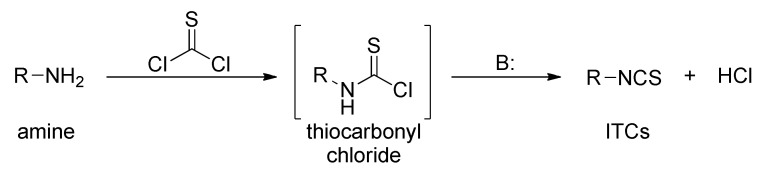
Synthesis of ITCs with thiophosgene.

The reaction of primary amines, or their salts with thiophosgene (**34**), is presently one of the oldest (but most commonly used) methods in the synthesis of ITCs [181,182]. The original product of the reaction is unstable thiocarbonyl chloride derivative [183], which, after elimination of hydrogen chloride, in an alkaline medium is transformed into the target isothiocyanate (Figure 9). Thiophosgene enables efficient conversion of both aliphatic and aromatic amines into ITCs. This method, however, is not suitable for bifunctional amines with reactive nucleophilic groups in the vicinal position because of subsequent cyclization of the original product.

A small excess of thiophosgene is recommended for use to prevent the formation of symmetrical thioureas as a side product. In contrast, the presence of an organic or inorganic base facilitates the formation of isothiocyanate and serves to neutralize hydrogen chloride. The use of concentrated strong bases (e.g., NaOH) is not recommended in this case because of the easy hydrolysis of ITCs in an alkaline medium. Reactions using thiophosgene can be carried out in a homogeneous—as well as a two-phase—system. In a two-phase system, water-organic solvents, both thiophosgene and the forming ITC, do not undergo subsequent reactions. Chloroform [184] and dichloromethane [185] are the most commonly used organic solvents and, as a base, calcium carbonate [186], sodium bicarbonate [187], or diluted sodium hydroxide solution [188] are usually used. The reaction is carried out at room temperature, and due to the heterogeneity system, intensive stirring is necessary [189]. In a homogeneous system, toluene [190] or acetone [191] are the most often used solvents, and triethylamine is used as a base.

**Figure 10:**
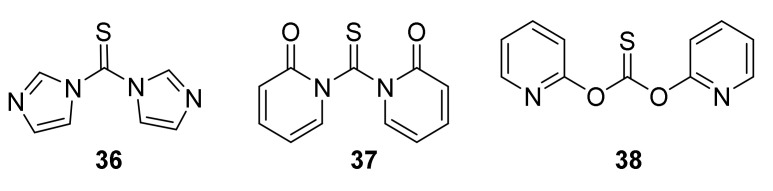
Thiophosgene surrogates.

Despite the relatively high toxicity of thiophosgene and its unpleasant smell, the aforementioned method is still popular due to its simplicity, versatility, and mild reactive conditions. For these reasons, thiophosgene is sometimes replaced by less toxic and less reactive reagents. Among them, the most widely used are thiocarbonyldiimidazole (**36**) [192], 1,1′-thiocarbonyldi-2,2′-pyridone (**37**) [193], and di-2-pyridyl thionocarbonate (**38**) [194] (Figure 10).

Although the use of these reagents guarantees high efficiency of thiocarbonylation, their use increases the cost-effectiveness of the method compared to the thiophosgene method.

### 3.2. Synthesis ITCs with a Desulfurizing Agent

**Figure 11:**
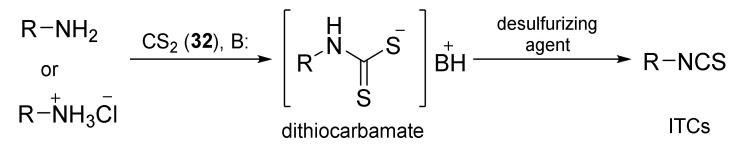
Synthesis of isothiocyanates using a desulfurizing agent.

The second most commonly used approach in the synthesis of ITCs is a two-step reaction, involving the conversion of amine or its salt and carbon disulfide (**35**) into the intermediate dithiocarbamate, which, in the presence of a desulfurizing reagent, undergoes transformation to the target ITCs [195] (Figure 11).

Most often, these two-stage reactions are performed in a “one-pot” version. The method is compatible with various functional groups and the range of its applicability is limited only by the nucleophilicity of the starting amines. In most cases, dithiocarbamates are formed quickly and in quantitative yield at room temperature. Only for aromatic amines, with strong electron-withdrawing substituents in the ring, does the formation of dithiocarbamates require the use of strong bases, such as sodium hydride, increasing the reaction time and elevating temperature. The above-mentioned method was described for the first time in 1886 by Hofmann [196]. Since then, there has been rapid development of ITC synthesis using various desulfurizing reagents. Unfortunately, the use of many of them involve drastic reaction conditions; byproducts formed in these reactions, such as heavy metal salts or carbodiimides, are difficult to remove [56]. Therefore, in recent years, effort has been made to find such desulfurizing reagents that would make in situ conversions of dithiocarbamates into the target ITCs quick, easy, and efficient.

**Figure 12:**
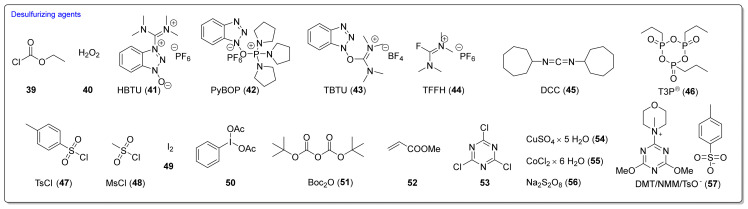
Selected desulfurizing agents.

Many desulfurizing reagents are known. Among them, the most widely used are ethyl chloroformate (**39**) [195,197], hydrogen peroxide (**40**) [198], peptide coupling reagents such as HBTU (**41**), PyBOP (**42**) [199], TBTU (**43**) [200], TFFH (**44**) [201], DCC (**45**) [202], and T3P^®^ (**46**) [203], tosyl chloride (**47**) [204], mesyl chloride (**48**) [205], molecular iodine (**49**) [206], with (diacetoxyiodo)benzene (**50**) [207], di-*tert*-butyl dicarbonate (**51**) [208], methyl acrylate (**52**) [209], 2,4,6-trichloro-1,3,5-triazine (**53**) [210], coper (II) sulfate (**54**) [211], cobalt (II) chloride (**55**) [212], sodium persulfate (**56**) [213], and 4-(4,6-dimethoxy-1,3,5-triazin-2-yl)-4-methylmorpholinium toluene-4-sulfonate (DMT/NMM/TsO^−^) (**57**) [214] (Figure 12). Eschliman and Bossmann [215] described some of the aforementioned reagents. 

The most commonly used desulfurizing reagents are briefly characterized below, because each of these reagents has different properties. Hydrogen peroxide (**40**) allows obtaining target alkyl ITCs in a short time at room temperature. The main disadvantages are the moderate yields and limitations to the synthesis of aliphatic ITCs. PyBOP (**42**) and TFFH (**44**) coupling reagents, in turn, enable the synthesis of ITC in the solid phase; however, the thiourea side-product is formed under the reaction’s conditions. The use of tosyl chloride (**47**) leads to aliphatic and aromatic ITCs in high yields. The use of di-*tert*-butyl dicarbonate (**51**), in the presence of a catalytic amount of DMAP or DABCO, allows obtaining aryl and aliphatic ITCs with high purity and in high yields within a few minutes. However, the synthesis of aryl ITC requires an extended reaction time. The most important advantage of this reagent is that the by-products formed in the reaction are easily removable gases (CO_2_, COS) or volatile liquids (*tert*-butyl alcohol). The application of (diacetoxyiodo)benzene (**50**) allows the preparation of aryl ITCs in high yields; however the high price of **50** precludes its use in a large-scale synthesis. For alkyl and cycloalkyl, ITCs yields are lower. Cheaper and non-toxic molecular iodine (**49**) also enables preparation of aromatic ITCs in high yields, and aliphatic isothiocyanates are formed in higher yields than in the reaction with (diacetoxyiodo)benzene (**50**). The reaction is environmentally friendly because it occurs in water in the presence of sodium bicarbonate [216]. The use of cyanuric chloride (**53**) allows obtaining high yields of aliphatic and aromatic ITCs, with both electron-donating and electron-withdrawing substituents. The advantage of this reaction is that it is performed in water. Propane phosphonic acid anhydride (T3P^®^) (**46**) enables synthesis of aliphatic, aromatic, and bifunctional ITCs, as well as isothiocyanates derived from esters of α-amino acids in high yields and with high purity. The method is compatible with a variety of protecting groups, and the reactions occur without racemization. A recently described coupling reagent 4-(4,6-dimethoxy-1,3,5-triazin-2-yl)-4-methylmorpholinium toluene-4-sulfonate (DMT/NMM/TsO^−^) (**57**) also enables synthesis of aliphatic and aromatic isothiocyanates, in a short time, in organic solvent as well as in water, and in microwave or in normal conditions, with very good yields. Additionally, DMT/NMM/TsO^−^ enables synthesis of isothiocyanate derivatives of natural and unnatural amino acids without racemization.

In addition to the synthesis of isothiocyanates performed under conventional conditions, microwave-assisted synthesis of ITCs using primary amines and carbon disulfide as substrates were recently developed [217]. This approach enables synthesis of aliphatic and aromatic ITCs in high yields. Transformation of intermediate dithiocarbamates into ITCs occurs without the addition of any desulfurizing agent.

### 3.3. Synthesis ITCs via the Tandem Staudinger/aza-Wittig Reaction

**Figure 13:**
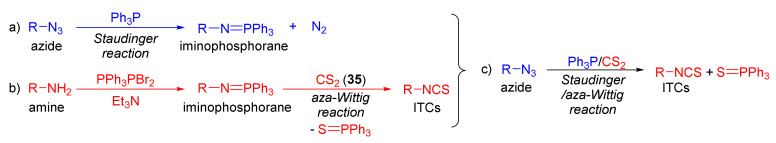
ITCs synthesis via Staudinger/aza-Wittig reaction.

The tandem Staudinger/aza-Wittig reaction is an alternative and equally efficient method used for the preparation of ITCs. The Staudinger reaction is the reaction of organic azide with a tricoordinated organophosphorus compound (triphenylphosphine (Ph_3_P), triphenyl phosphite, or triethyl phosphite) leading an iminophosphorane with the losses of nitrogen (Figure 13a) [218].

The use of the aza-Wittig reaction for ITC synthesis was first reported by Molina et al. [219]. It involves the conversion of primary amines and dibromotriphenylphosphorane (triphenylphosphine dibromide) (PPh_3_PBr_2_) into iminophosphoranes, followed by their reactions, with carbon disulfide (**35**) giving target ITCs (Figure 13b). The tandem Staudinger/aza-Wittig reaction was first described by Tsuge et al. [220] in 1984 (Figure 13c). The advantage of the tandem reaction over the two-stage process, and the mechanistic aspects of this transformation were reported in 2006 by Isoda et al. [221]. In a tandem reaction, iminophosphorane formed from azide and Ph_3_P reacts in situ with CS_2_, “giving” the target ITCs in high yield (Figure 13c). However, problems may occasionally occur with the separation of ITCs from an equimolar amount of triphenylphosphine sulfide formed in the reaction, and small amounts of triphenylphosphine oxide (a by-product of Ph_3_P oxidation).

### 3.4. The Latest Approaches to ITC Syntheses

**Figure 14:**
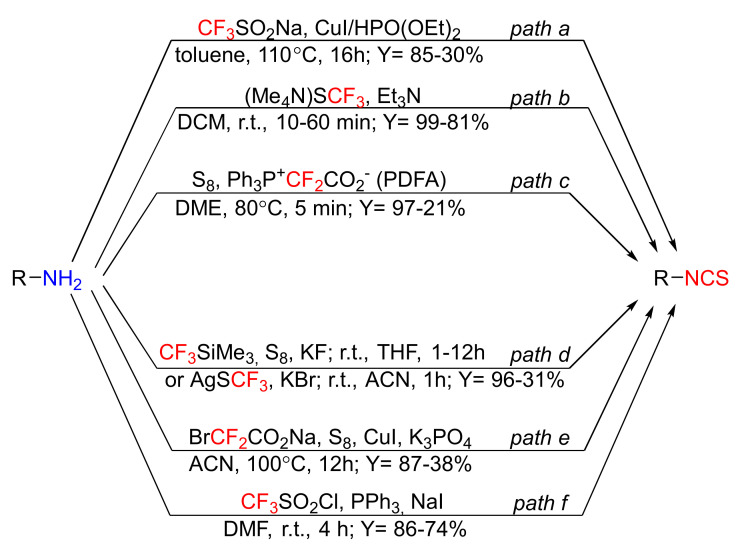
Synthesis of ITCs using fluorine-containing reagents.

In the past few years, several efficient approaches to the syntheses of ITCs were developed. They utilize primary amines as starting materials, and the appropriate fluorine reagents as desulfurizing agents, replacing toxic thiophosgene or carbon disulfide (Figure 14).

Liao et al. [222] used primary amines as substrates, and the Langlois reagent (CF_3_SO_2_Na) [223] in the presence of copper iodide and diethyl phosphonate, to obtain a library of structurally diverse aromatic as well as aliphatic ITCs in high yields (Figure 14, *path a*). The replacement of toxic thiophosgene with the Langlois reagent resulted in the development of a simple, safe, and environmentally friendly method of ITC synthesis. The disadvantages of this method include long reaction times, high temperatures, and low functional group tolerance (e.g., pyridinyl). 

A highly efficient, selective, and fast method for the synthesis of ITCs from primary amines, by using a bench-stable, solid reagent (Me_4_N)SCF_3_ (tetramethylammonium trifluoromethane thiosulfate), was described by Schoenebeck et al. [224] (Figure 14, *path b*). The target ITCs are easily separated from the solid side products, and the method is compatible with several functional groups. In addition, the use of (Me_4_N)SCF_3_ enables the transformation of secondary diamines into cyclic thioureas.

An alternative method of ITC preparation involves the reaction of thiocarbonyl fluoride (CF_2_=S), generated from difluorocarbene and elemental sulfur (S_8_), with primary amines (Figure 14, *path c*) [225]. Difluorocarbene is valuable and versatile, intermediate in organic synthesis, and is generated from Ph_3_P^+^CF_2_CO_2_^−^ (PDFA) [226]. Synthesis of ITCs is very fast, and the series of aromatic isothiocyanates with electron-donating and electron-withdrawing groups in the aromatic ring, as well as aliphatic ITCs, have been obtained in high yields. The only disadvantage of this method is that PDFA is relatively expensive, and in the reaction of *o*-phenylenediamines with the PDFA/S_8_ system, difluoromethylthiolated heterocycles are formed. 

The methodology using thiocarbonyl fluoride was also reported by Zhen et al. [227]. The authors obtained a pool of aromatic and aliphatic ITCs, in moderate to good yields, in the reaction of thiocarbonyl fluoride generated from CF_3_SiMe_3_ (the Ruppert–Prakash reagent), elemental sulfur, and KF or AgSCF_3_ at mild conditions (Figure 14, *path d*). The Ruppert–Prakash reagent is a stable, relatively cheap, easy to handle and widely used reagent [228].

In 2019, Feng and Zhang [229] established an organophosphine-free, one-pot, copper-catalyzed, three-component synthesis of ITCs from primary amines, sodium bromodifluoroacetate (BrCF_2_CO_2_Na), and elemental sulfur (S_8_) in the presence of K_3_PO_4_, as a base (Figure 14, *path e*). According to the authors, isothiocyanation of amines takes place through the intermediate thiocarbonyl fluoride, or alternatively, via intermediate isocyanide, followed by its reaction with sulfur—this despite the fact that the reaction requires a prolonged time and a high temperature for completion, compatible with different functional groups, and a series of aromatic as well as aliphatic isothiocyanates have been obtained in moderate to good yields. For *o*-phenylenediamine and *o*-hydroxyaniline, as substrates, the appropriate 1-difluoromethyl benzimidazole and benzoxazole have been obtained, respectively. This and other methods of synthesis isothiocyanates using element sulfur have been described in a recently published review article [230].

Recently, Wei et al. [231] published an efficient method synthesis of a library of isothiocyanates from primary amines using trifluoromethanesulfonyl chloride in the presence of reducing the agent triphenylphosphine and sodium iodide (Figure 14, *path f*). Trifluoromethanesulfonyl chloride (CF_3_SO_2_Cl) is commercially available, cheap, easy to handle, and widely used (e.g., in electrophilic chlorination [232], trifluoromethylation [233], or in chloro-trifluoromethylthiolation of alkenes and alkyne [234]) reagents. The authors obtained aromatic ITCs with electron-donating or electron-withdrawing groups as well as aliphatic ITCs with good yields. Except for ITCs, the authors synthesized thiocarbamoyl fluorides using secondary amines with good yields.

## 4. Synthesis of Bifunctional Analogs of Sulforaphane and Their Properties

This section focuses on the most commonly used methods for the synthesis of SFN and its difunctional analogues. This involves isothiocyanation of amines with thiophosgene or carbon disulfide/desulfurizing agents system, or the tandem Staudinger/aza-Wittig method using azides as substrates. The methods where SFN and its analogues are obtained by myrosinase catalyzed hydrolysis of glucosinolates isolated from Cruciferae [235,236,237], or obtained by other methods (e.g., reaction with KSCN [238]), are not included. The choice is restricted to SFN and its natural or synthetic bifunctional analogues with an unbranched alkyl chain containing two to six carbon atoms and having sulfinyl, sulfanyl, sulfonyl, phosphonate, phosphinate, phosphine oxide, carbonyl, ester, amide, ether, or a second isothiocyanate group. Except for the synthesis, the biological activity of SFN and its analogues are also discussed.

### 4.1. Synthesis of Sulforaphane and Its Sulfur Analogues and Their Properties

**Figure 15:**
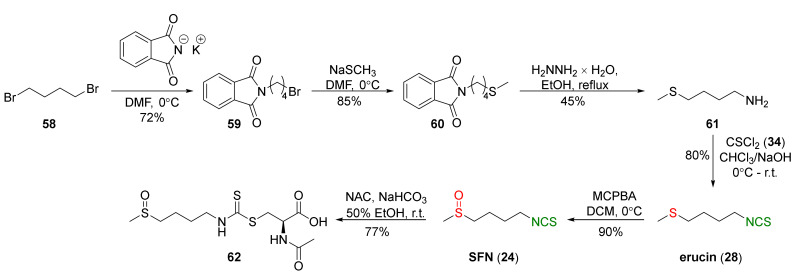
Synthesis of SFN (**24**) and its mercapturic acid **62**.

One of the most commonly used synthetic pathways of SFN (**24**) utilizing thiophosgene (**34**) for isothiocyanation of amines was described by Vermeulen et al. [239]. The authors started from 1,4-dibromobutane (**58**) and potassium phthalimide to form 1-bromo-4-*N*-phthalimido)butane (**59**). Next, compound **59** was converted into *N*-(4-methylsulfanyl-butyl)phthalimide (**60**) in the reaction with sodium methyl mercaptide. Deprotection of the amino group in **60** with hydrazine hydrate provided 4-(methylsulfanyl)butan-1-amine (**61**), a key intermediate in the synthesis of SFN and its analogues. Thus, erucin (**28**) was obtained in an 80% yield by the reaction of amine **61** with thiophosgene (**34**) in a two-phase system (chloroform/water) using sodium hydroxide as a base. Oxidation of **28** with *m*-chloroperbenzoic acid (MCPBA) afforded SFN (**24**) in a 90% yield. In the final stage, the authors converted SFN (**24**) into the *N*-acetyl-*S*-(*N*-4-methylsulfinylbutylthiocarbamoyl)-L-cysteine (sulforaphane mercapturic acid, **62**) in the reaction with *N*-acetyl-L-cysteine (NAC) in a 77% yield (Figure 15).

**Figure 16:**
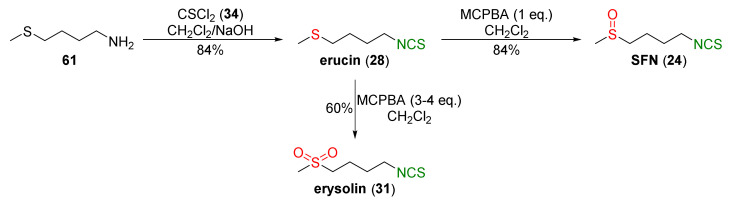
Synthesis of erucin (**28**), SFN (**24**), and erysolin (**31**).

The same approach to SFN preparation was used by Mays et al. [240]. The authors transformed 4-(methylsulfanyl)butan-1-amine (**61**) into erucin (**28**) in a 84% yield using thiophosgene (**34**) and sodium hydroxide as a base. Oxidation of **28** with an equimolar amount of MCPBA resulted in SFN (**24**) in a 84% yield, and the use of excess MCPBA led to erysolin (**31**) in a 60% yield (Figure 16).

The cytotoxicity of erucin, SFN and erysolin, as well as other synthesized ITCs, were examined on eight human cancer cell lines representing a broad range of carcinomas, including breast, colon, CNS, livery, ovary, prostate, and a mouse mammary normal epithelial cell (NmuMG) control line (Table 1).

SFN (**24**) had the lowest IC_50_ values for Hep3B (human liver carcinoma), erucin (**28**) for SF-268 (human CNS glioblastoma), and erysolin (**31**) for MCF7 (women’s breast cancer ER+ (Luminal A). SFN and erysolin presented the highest IC_50_ for NCI/ADR RES (human breast carcinoma), and erucin for HCI-H460 (human breast carcinoma). IC_50_ on NmuMG for SFN and erysolin was almost similar; however, for erucin, it was unquestionably higher (Table 1).

**Figure 17:**
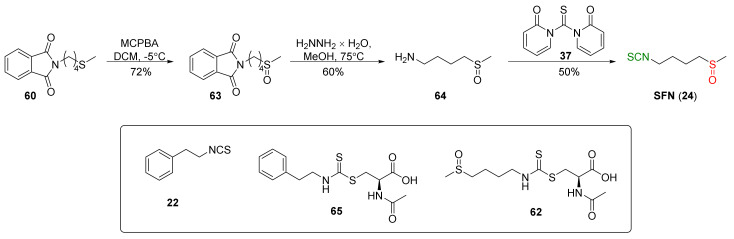
Synthesis of SFN using 1,1′-thiocarbonyldi-2,2′-pyridone (**37**).

In addition to thiophosgene, its substitute (1,1′-thiocarbonyldi-2,2′-pyridone (**37**)) was used for SFN synthesis. Conaway et al. [241] oxidized starting *N*-(4-methylsulfanyl-butyl)phthalimide (**60**) to *N*-(4-methylsulfinyl-butyl)phthalimide (**63**) by MCPBA, followed by the amino group deprotection in **63** with hydrazine monohydrate to obtain 4-(methylsulfinyl)butan-1-amine (**64**) in a 60% yield. In the final step, isothiocyanation of amine **64** with **37** afforded SFN (**24**) in a 50% yield (Figure 17).

The authors tested tumor-inhibitory activities of phenethyl isothiocyanate (PEITC, **22**), SFN (**24**), and their *N*-acetylcysteine conjugates (**65** and **62**) (Figure 17) on the development of malignancy from benign tumors in the lung of A/J mice after administration of NNK and B(*a*)P, two potent carcinogens of cigarette smoke involved in lung cancer in smokers. The results show that PEITC, SFN, and their *N*-acetylcysteine conjugates added to the diet after lung adenomas, inhibiting the progression to adenocarcinomas. The inhibitory effects of these compounds are likely to be associated with a combination of reduced cell proliferation and induced apoptosis.

**Figure 18:**
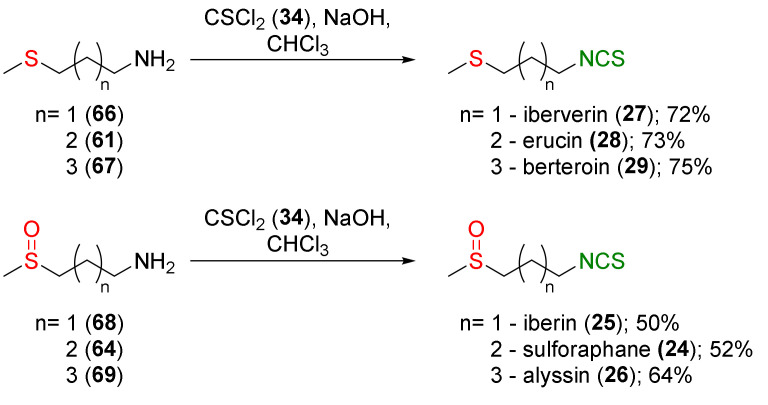
Synthesis SFN (**24**), erucin (**28**), and their analogs using thiophosgene.

SFN (**24**), its homologues iberin (**25**), and alyssin (**26**), as well as erucin (**28**), iberverin (**27**), and berteroin (**29**) were synthesized by Moon and co-workers [242]. The authors converted difunctional amines with sulfanyl (**61**, **66** and **67**) and sulfinyl moieties (**64**, **68** and **69**) and with unbranched alkyl chains containing 3 to 5 carbon atoms into the corresponding ITCs (**24**–**29**), using thiophosgene (**34**) and sodium hydroxide as a base, with moderate to good yields (Figure 18).

The bactericidal activity against *H. pylori* of synthesized ITCs was tested. All tested ITCs (**24**–**29**) showed strong anti-*Helicobacter* activity at the level of a 5 mg/disk exhibiting >5 cm inhibitory zones. 

**Figure 19:**
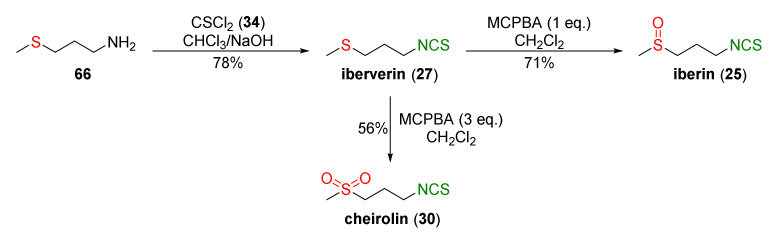
Synthesis of iberverin (**27**), iberin (**25**), and cheirolin (**30**).

Based on the methodology presented in Figure 15, Ernst and co-workers [243] obtained iberverin (**27**) from 4-(methylsulfanyl)propan-1-amine (**66**) and thiophosgene (**34**) in a 78% yield. Oxidation of iberverin (**27**), with equimolar or excess amounts of MCPBA, provided iberin (**25**) or cheirolin (**30**) with 71% and 56% yields, respectively (Figure 19).

It was found that iberin (**25**), iberverin (**27**), and cheirolin (**30**) significantly induced Nrf2 nuclear translocation in NIH3T3 fibroblasts. The increase of nuclear Nrf2 levels was accompanied by an increase of heme oxygenase (HO-1) and γ-glutamylcysteine synthetase (γGCS) mRNA and other protein levels. Iberverin (**27**), iberin (**25**), and cheirolin (**30**) exhibited a similar potency to SFN (**24**) in terms of their Nrf2-dependent gene expression. Induction of Nrf2 by iberverin, iberin, and cheirolin may have occurred via the extracellular signal-related kinase (ERK)-dependent signal-transduction pathway.

**Figure 20:**
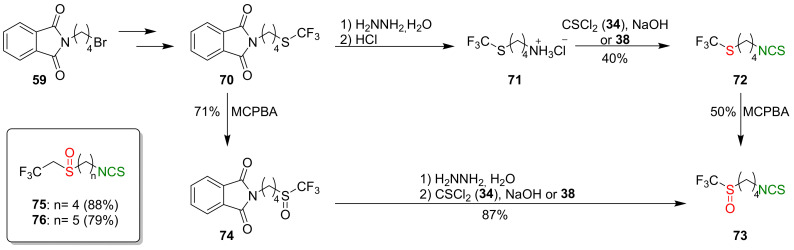
Synthesis of trifluoromethyl (**73**) and trifluoroethyl (**75**–**76**) analogs of SFN.

A large group of ITCs can be considered as SFN analogues, having non-methyl substituents on the sulfinyl group. These compounds include fluorine derivatives of SFN, synthesized by Kiełbasiński and co-workers [244]. Thus, substrate **59** was converted into a trifluoromethyl derivative **70**, and transformed the target product **73** via two independent routes. In the first one, amine hydrochloride **71**, obtained after deprotection of amino group of compound **70**, was converted into isothiocyanate **72** with a yield of 40% by the reaction with thiophosgene (**34**) or di-2-pyridyl thiocarbonate (**38**). Oxidation of the trifluoromethylsulfanyl group of **72**, using MCPBA, gave the trifluoromethyl analog of SFN **73** a 50% yield. In the second synthetic pathway, phthalimido-sulfide **70** was oxidized to sulfoxide **74**, followed by hydrazinolysis and subsequent isothiocyanation; thus, forming amine hydrochloride with thiophosgene (**34**) to give the target isothiocyanate **73** in a 87% yield. Using the first synthetic route, the authors also obtained trifluoroethyl analogues of SFN **75** and alyssin **76** with high yields (Figure 20).

The ITCs **73**, **75**–**76**, obtained as racemates, were separated on preparative chiral HPLC to the enantiomerically pure products. All three pairs of enantiomers of fluorine-containing analogs **73**, **75**–**76** were tested in vitro for their cytotoxicity against malignant melanoma cell lines Malme-3M and normal skin fibroblast Malme-3. In Table 2, the activity of the most promising compound **75** is presented.

After 48 h of incubation, the optically active fluorine analogs (*R*)-**75** and (*S*)-**75** exhibited higher cytotoxicity than SFN; however, after 72 h of incubation, the cytotoxicity was comparable. These results could suggest that the anticancer mechanism of SFN and its fluorine analogs are different. As seen from Table 2, the most promising was (*S*)-1-isothiocyanato-4-((2,2,2-trifluoroethyl)sulfinyl)butane (*S*)-**75**).

**Figure 21:**
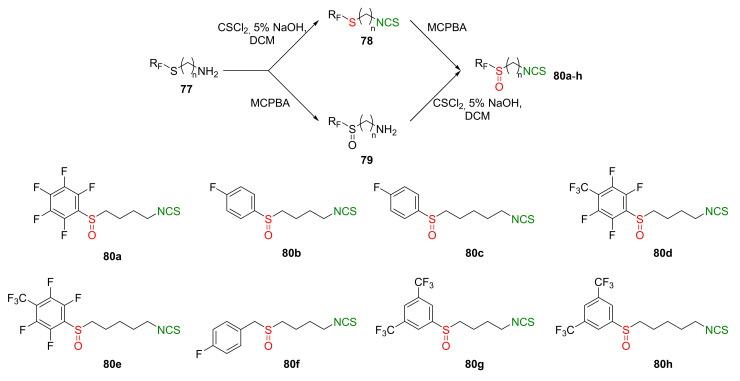
Synthesis of fluoroaryl and fluoroarylmethyl analogs **80a**–**h**.

Recently, the same research group described synthesis and biological activity of fluoroaryl analogs of SFN with 4 and 5 carbon atoms in the unbranched alkyl chain [245]. Thus, the starting ω-aminoalk-1-yl fluoroaryl or fluoroarylmethyl sulfides **77** were converted to the final fluoroaryl or fluoroarylmethyl analogs of SFN **80a**–**h** by two alternative pathways (Figure 21). In the first path, amines **77** were transformed in the reaction with thiophosgene (**34**) into fluoroaryl or fluoroarylmethyl ω-isothiocyanatoalk-1-yl sulfides **78**, followed by oxidation with MCPBA, to afford the target isothiocyanates **80a**–**h**. In the second approach, sulfides **77** were oxidized to sulfoxides **79**, followed by the treatment with thiophosgene, to provide **80a**–**h**. Final α-(fluorosulfinyl)- and α-(fluoroarylmethylsulfinyl)-ω-isothiocyanatoalkanes **80a**–**h** were obtained in good and very good yields.

All fluoroaryl and fluoroarylmethyl analogs **80a**–**h** were tested in vitro for their anticancer, antibacterial, antifungal, and antiviral properties. The anticancer activity on the skin cancer cell line (MALME-3M), colon cancer cell line (HT-29), and breast cancer cell lines (MCF-7 and MDA-MB-231), as well as their normal cell lines, were studied. After 72 h of incubation, all compounds presented higher activity than SFN. The most active on all cancer cell lines were ITCs **80d** and **80e** (Table 3). 

It was found that fluoroaryl or fluoroarylmethyl analogs **80a**–**h**, as well as SFN, were inactive against Gram-negative bacteria (*E. coli* and *P. aeruginosa*). However, SFN and its fluoroaryl or fluoroarylmethyl analogs **80b**–**f** had antibacterial activity against Gram-positive bacteria, including methicillin-resistant *S. aureus* (MARS), except for *B. subtilis* and *E. hirae* strains. In particular, ITC **80e** was the most active (MIC values were in the range of 0.031–0.0625). Concerning anti-HIV activity, only ITC **80e** showed similar activity to SFN (0.5 μM inhibited HIV replication in 9% of cases). Other ITCs were inactive.

**Figure 22:**
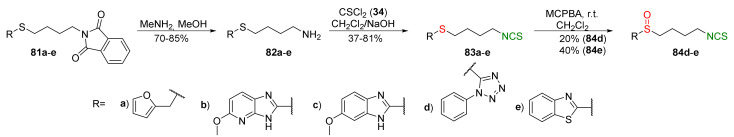
Synthesis of heterocyclic analogues **84d**–**e** of SFN.

In 2016, Shi et al. [246] synthesized a series of analogues of SFN, in which the methyl group adjacent to sulfur was replaced with heterocyclic moieties, such as furan (a), 5-methoxy-*3H*-imidazo[4,5-b]pyridine (b), 6-methoxy-*1H*-benzo[d]imidazole (c), 5-phenyl-*1H*-tetrazole (d), or benzo[d]thiazole (e). The sulfides **81a**–**e** were transformed into amines **82a**–**e**, and then were converted into sulfide derivatives of ITCs **83a**–**e** with thiophosgene (**34**), in good and very good yields. In the next step, compounds **83d**–**e** were oxidized with MCPBA into sulfoxide analogues of SFN **84d**–**e** with good yields (Figure 22).

**Figure 23:**
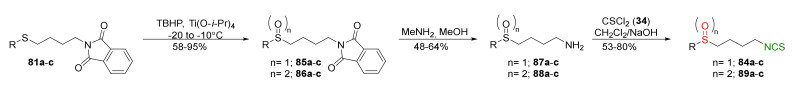
Synthesis of sulfoxide **84a**–**c** and sulfone **89a**–**c** analogues of SFN.

Sulfoxide and sulfone analogues of SFN with heterocyclic moieties **84a**–**c** and **89a**–**c** were prepared in a modified way. Compounds **81a**–**c** were oxidized by *tert*-butyl hydroperoxide (TBHP) to sulfoxides **85a**–**c** and sulfones **86a**–**c** and then transformed to amines **87a**–**c** and **88a**–**c** using methylamine. In the final step, isothiocyanation of amines **87a**–**c** and **88a**–**c** with thiophosgene afforded ITCs **84a**–**c** and **89a**–**c** in good yields (Figure 23).

**Figure 24:**
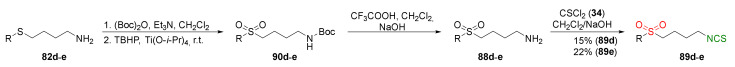
Synthesis of sulfone analogues of SFN **89d**–**e**.

Sulfone analogues with tetrazole and thiazole moieties **89d**–**e** were obtained from amines **82d**–**e** prior to Boc protection of amino groups, and oxidation formed *N*-Boc amines to sulfone analogues **90d**–**e**. Removal of the Boc protecting group in **90d**–**e**, followed by the reaction free amines **88d**–**e** with thiophosgene, allowed to obtain the final sulfone analogues **89d**–**e** in low yields (Figure 24).

The series of SFN analogues with heterocyclic moieties **83a**–**e**, **84a**–**e**, and **89a**–**e** were evaluated for their anticancer activities (breast cancer cell lines MCF-7 and SUM-159, acute leukemia stem cell-like cell line, and KG-1a). Among all synthesized analogues, tetrazole analogues **83d**, **84d**, and **89d** were generally the most potent—and significantly more active—than SFN against the tree cancer cell lines (Table 4).

Moreover, compound **83d**, as well as SFN, induced apoptosis in the SUM-159 cell line by increasing caspase-3 activity and significantly reducing the ALDH+ subpopulation in the SUM-159 cell line from 3.10% to 0.16%.

**Figure 25:**
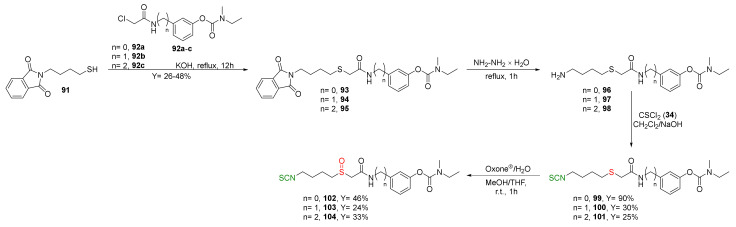
Synthesis of erucin and SFN-rivastigmine hybrids **99**–**104**.

Recently Sestito et al. [247] designed and synthesized a new class of multitarget H2S-donor hybrids **99**–**104**, combining the rivastigmine-scaffold, an acetylcholinesterase inhibitor with brain region selectivity [248], a well-known drug approved for Alzheimer’s disease, with SFN and erucin. Thus, mercaptobutyl derivative **91** was alkylated with the appropriate chloroacetamide **92a**–**c** under the basic conditions to give the corresponding thioethers **93**–**95** with moderate yields. Hydrazinolysis of derivatives **93**–**95** provided amines **96**–**98**, which, after the reaction with thiophosgene, gave ITCs **99**–**101** in 25–90% yields. Oxidation of ITCs **99**–**101** by Oxone^®^ led to sulfoxide **102**–**104** in moderate yield (Figure 25).

Studies on the murine microglia cell line (BV-2) showed that all synthesized ITC hybrids **99**–**104** exhibited a H_2_S-donor profile in vitro. Compounds **99**–**104** showed significantly anti-inflammatory and antioxidant activities and induced the expression of proteins (i.e., GSH) involved in the antioxidant defense in the neuronal cell line. All hybrids produced a significant decrease in ROS production elicited by pro-inflammatory stimulus compared to the rivastigmine, which completely lacks antioxidant activity. The new hybrids were also able to reduce NO release in BV-2 cells, whereas rivastigmine showed no effect. Moreover, the most active compounds **99** and **100** increased the GSH level in the human neuroblastoma cell line SH-SY5Y.

**Figure 26:**
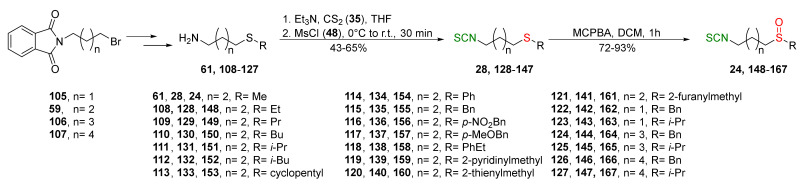
Synthesis of SFN (**24**) and its derivatives **148**–**167**.

In 2013, Hu et al. [249] obtained an extensive library of sulfanyl and sulfinyl analogs of SFN with an alkyl or phenyl substituent on a sulfur atom, and with an alkyl linker containing 3 to 6 carbon atoms using the dithiocarbamate approach and applying mesyl chloride (**48**) as a desulfurizing agent. Sulfide derivatives of ITCs **28**, **128**–**147**, were prepared, in the presence of Et_3_N, from the corresponding ω-(alkylthio)alkanamines **61**, **108**–**127**, carbon disulfide (**35**), and mesyl chloride (**48**) in 43–65% yields. The key intermediates, the appropriate ω-(alkylthio)alkanamines **61**, and **108**–**127**, were obtained from ω-bromoalkylphthalimides **59** and **105**–**107** in a standard procedure. Oxidation of sulfides **28**, and **128**–**147** with MCPBA, led to sulfoxide analogues **24** and **148**–**167**, with very good yields (Figure 26).

All synthesized ITCs **28**, **24**, **148**–**167** were evaluated in vitro for their cytotoxicity against liver hepatocellular carcinoma (HepG2), human lung adenocarcinoma (A549), woman breast cancer ER+ (Luminal A)(MCF-7), human colon cancer cell line (HCT-116) and human neuroblastoma cell line (SH-SY5Y). All tested compounds exhibited more potent inhibitory against five cancer cell lines than SFN. The IC_50_ for the most active ITCs are presented in Table 5.

For HepG2, the most active was ITC **151** with the IC_50_ value 2.05 μM. ITC **161** had the strongest inhibition against A549 with IC_50_ 5.64 μM, and ITC **155** possess significant cytotoxicity for MCF-7 with IC_50_ 3.3 μM. Derivative **152** against HCT-116 had an IC_50_ value of 2.06 μM, and isothiocyanate **157** against SH-SY5Y ITC had an IC_50_ value 2.79. Based on the tested ITCs **28**, **24**, **148**–**167**, it was found that compounds with sulfanyl moiety **135** showed weaker inhibitory effects than most derivatives; however, compound **168** with a sulfone group showed a higher inhibitory activity against all cancer cell lines than SFN. It may indicate that replacing the sulfoxide group with a sulfone group results in higher biological activity. The studies on anticancer mechanisms examined on the HepG2 cancer cell line showed that SFN, as well as model ITC **155**, could induce the S or G_2_/M phase cycle arrest, and **155** has more potent inhibitory activity than SFN. Moreover, ITC **155** exhibited greater induction of apoptosis upon treatment than SFN. ITC **155** presented a time- and dose-dependent activation on the Nrf2 transcription factor. Moreover, **155** acted as a more potent Nrf2 inducer than SFN.

**Figure 27:**
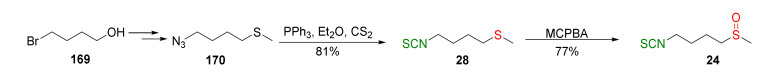
Synthesis of SFN and erucin via Staudinger/aza-Wittig reaction.

Meijer and co-workers [163] synthesized SFN (**24**) and its analogue erucin (**28**) via the tandem Staudinger/aza-Wittig reaction. Thus, the starting 4-bromo-butanol (**169**) was transformed using a standard procedure into azido thioether **170**. Then, this compound, in the reaction with triphenylphosphine and carbon disulfide, was converted into erucin (**28**) in an 81% yield. SFN (**24**) was obtained by oxidation of erucin (**28**) with MCPBA in 77% yield (Figure 27).

Studies on *P. aeruginosa* show that SFN and erucin strongly inhibit quorum sensing (QS) and virulence (biofilm formation and pyocyanin production). The assays on *P. aeruginosa* and in *E. coli* strongly indicate that SFN and erucin effectively bind LasR, resulting in inhibition of QS activation at concentrations that can be found in broccoli.

**Figure 28:**
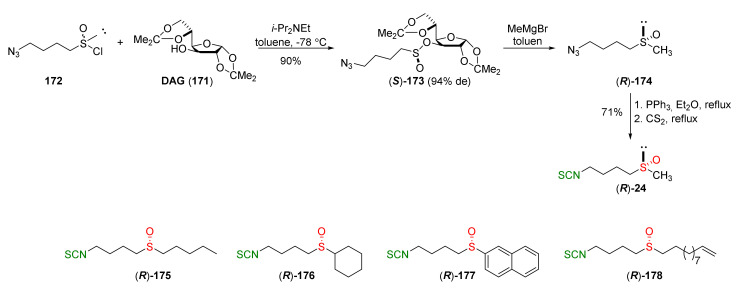
Synthesis of enantiopure (*R*)-SFN ((*R*)-**24**) and its enantiopure **175**–**178** analogues.

The Staudinger/aza-Wittig tandem reaction was used in the synthesis of enantiomerically pure (*R*)-SFN ((*R*)-**24**) [250] using diacetone-D-glucofuranose (**171**) (DAG)-methodology [251]. The stereoselective synthesis of SFN was based on the reaction of 1-azidobutanesulfinyl chloride (**172**) with DAG (**171**), using Hünig’s base as a catalyst, and affording the sulfinate ester (*S*)-**173** in a 90% yield and in 94% diastereomeric excess. Reaction of methylmagnesium bromide with the sulfinate ester (*S*)-**173** provided 4-azidobutyl methyl sulfoxide ((*R*)-**174**) with inversion of configuration, which, in turn, under the Staudinger/aza-Wittig reaction with triphenylphosphine and carbon disulfide, gave enantiopure (*R*)-SFN ((*R*)-**24**) in a 71% yield. The same methodology was applied in the synthesis of the other analogues. Thus, condensation of the selected Grignard reagents with sulfinate ester (*S*)-**173** led to the desired azido sulfoxides, which, in a two-step Staudinger/aza-Wittig reaction, allowed obtaining enantiopure (*R*)-sulfinyl ITCs **175**–**178** in 47–90% yields (Figure 28).

The enantiopure analogues of SFN **175**–**178** were assayed in the activation of the cytoprotective transcription factor Nrf2. The obtained results indicate that there is a close relationship of Nrf2 activation and the steric demand of the substituent at the sulfinyl sulfur. The SFN analogues with an alkyl side chain were more active than the analogue with the aromatic sulfinyl group (*R*)-**177**. Within the dialkyl sulfoxides (*R*)-**175**, (*R*)-**176**, and (*R*)-**178**, the most active one is the pentyl sulfoxide (*R*)-**175**, while the dialkyl sulfoxide (*R*)-**178**, with an extended alkyl chain, is the least active.

**Figure 29:**
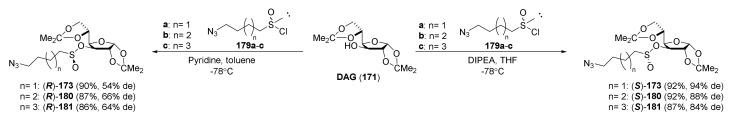
Enantiodivergent synthesis of (*S*)- and (*R*)-sulfinate esters **173**, **180**, and **181** using the DAG-methodology.

The same research group in 2014 described synthesis of both enantiomers of SFN homologues with alkyl chains of different lengths between the isothiocyanate group and the sulfinyl group, with the chiral auxiliary derived from diacetone-D-glucofuranose (DAG) [252]. Thus, azidoalkanesulfinyl chlorides **179a**–**c** in reaction with diacetone-D-glucofuranose (**171**), in the presence of DIPEA as a base, afforded the (*S*)-sulfinate esters **173** and (*S*)-**180**–**181** with high yields, and good diastereomeric excesses. However, when pyridine was used as a base, (*R*)-sulfinate esters **173** and (*R*)-**180**–**181** were obtained in high yields, although in lower diastereomeric excesses (Figure 29).

**Figure 30:**
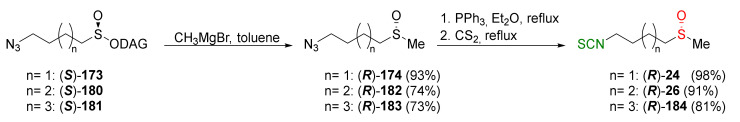
Synthesis of enantiopure (*R*)-SFN ((*R*)-**24**), (*R*)-alyssin ((*R*)-**26**), and their homologue *(R*)-**184**.

The replacement of chiral auxiliary in sulfinates **173** and **180**–**181** by the Grignard reagents takes place with the inversion of configuration at the sulfinyl sulfur. Condensation of methylmagnesium bromide with the sulfinate ester (*S*)-**173**, (*S*)-**180**, and (*S*)-**181** afforded the corresponding azidoalkyl methyl sulfoxides (*R*)-**174**, (*R*)-**182**, and (*R*)-**183** in high yields. Subsequent reactions of azides with triphenylphosphine and carbon disulfide led to enantiomerically pure (*R*)-SFN ((*R*)-**24**), (*R*)-alyssin ((*R*)-**26**), and their homologue (*R*)-**184** with high yields (Figure 30).

**Figure 31:**
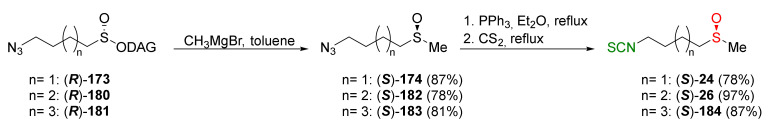
Synthesis of enantiopure (*S*)-SFN ((S)-**24**), (*S*)-alyssin ((*S*)-**26**), and their homologue *(S*)-**184**.

Using the same methodology, the enantiomerically pure (*S*)-SFN ((*S*)-**24**), (*S*)-alyssin ((*S*)-**26**), and their homologue (*S*)-**184** were obtained from the sulfinate ester (*R*)-**173**, (*R*)-**180** and (*R*)-**181** with high yields (Figure 31).

**Figure 32:**
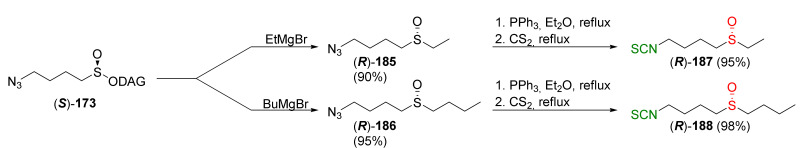
Synthesis of enantiopure analogues of (*R*)-SFN-(*R*)-**187** and (*R*)-**188**.

In the same way, using ethylmagnesium bromide or butylmagnesium bromide as a Grignard reagent, the authors synthesized analogues of SFN, in which the methyl group was replaced by ethyl or butyl substituents. Enantiopure sulfinyl analogues (*R*)-**187** and (*R*)-**188** were prepared with high yields (Figure 32).

The efficiency of the synthesized compounds as the inductors of phase II detoxifying enzymes was evaluated by studying their ability to activate the cytoprotective transcription factor Nrf2. It was shown that homologues containing 5 and 6 carbon atoms in the alkyl chain showed beneficial effects on the activation of Nrf2, increasing the steric size of the substituent on the sulfur has negative effects on the biological activity. Which sulfur stereochemistry has no effect on the ability of these analogues to activate the cytoprotective transcription factor Nrf2 is additionally noted (Table 6).

All synthesized (*R*) and (*S*) analogues of SFN were evaluated in vitro for their cytotoxicity against human lung adenocarcinoma (A549) and the fetal lung fibroblast (MRC-5) cell line as normal cells. Synthesized analogues were more effective against lung cancer than (*R*)-SFN, and among them, (*S*)-**184** was the most promising compound that showed slight selectivity than SFN towards the cancer cell (Table 6).

### 4.2. Synthesis of Phosphorus Analogues of Sulforaphane and Their Properties

In this section, phosphorus analogues of sulforaphane (P-SFN) compounds, where methylsulfinyl moiety was replaced by phosphonate, phosphinate, or phosphine oxide groups, are described.

**Figure 33:**
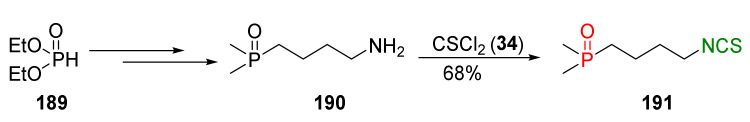
Synthesis of (4-isothiocyanatobutyl)dimethylphosphine oxide (**191**).

The first type of information about phosphorus analogues of SFN came from Posner et al. [188]. The synthesis and biological activity of (4-isothiocyanatobutyl)dimethylphosphine oxide (**191**), the phosphine oxide analogue of SFN, was described. The target (4-isothiocyanatobutyl)dimethylphosphine oxide (**191**) was obtained under basic conditions by isothiocyanation of (4-aminobutyl)dimethylphosphine oxide (**190**) with thiophosgene (**34**) in a 68% yield (Figure 33). The key intermediate **190** was prepared via a standard procedure for the synthesis of phosphine oxides from diethyl phosphite (**189**).

Phosphorus analogues of SFN **191**, as well as sulforaphane (**24**), were evaluated in vitro as inducer potencies of NAD(P)H:quinine oxidoreductase (QR) in murine hepatoma cells (Hepa 1c1c7) (Table 7).

As seen in Table 7, the potency of dimethylphosphine oxide **191** and SFN (**24**) were almost equal.

**Figure 34:**
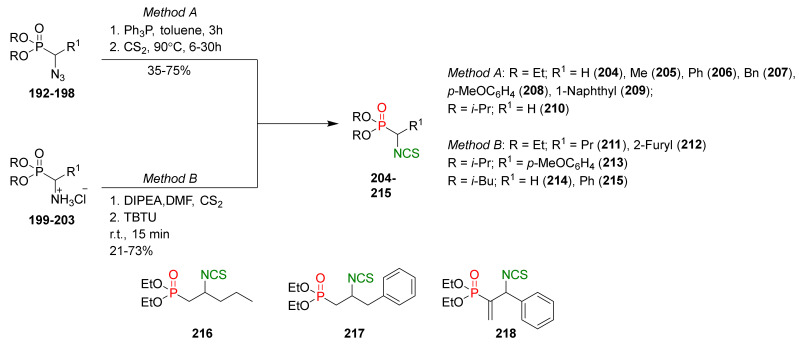
Synthesis of α- and β-dialkoxyphosphoryl isothiocyanates **204**–**218**.

In 2011, Oleksyszyn et al. [200] described the synthesis of a series of α- and β-dialkoxyphosphoryl isothiocyanates. Dialkyl α-(isothiocyanatoalkyl)phosphonates **204**–**215** were obtained in two ways, as shown in Figure 34. In the first way (method A) for the transformation of dialkyl α-azidoalkylphosphonates **192**–**198** into isothiocyanates **204**–**210**, the tandem Staudinger/aza-Wittig reaction was used, and the final P-SFN were obtained in 35–75% yields. In the second way (method B), the targets P-SFN **211**–**215** were prepared with good and very good yields via desulfuration of dithiocarbamates formed in situ from aminophosphonates hydrochlorides **199**–**203** and carbon disulfide with TBTU. Method B was also used for the synthesis of β-dialkoxyphosphoryl isothiocyanates **216**–**218** (Figure 34).

The obtained P-SFN were tested for cytotoxicity on five cancer lines: lung cancer (A549), breast cancer (T47D and MCF-7), colon cancer (LoVo), as well as its doxorubicin-resistant variant–LoVo/DX. All tested α- and β-dialkoxyphosphoryl isothiocyanates showed very good antiproliferative activities in vitro comparable to the most active of natural isothiocyanates BITC (**21**) and PEITC (**22**). Moreover, the mechanism of anticancer activity was evaluated. Isothiocyanate **205** showed inhibition of the cell cycle in the subG_0_/G_1_ phase, and compound **216** inhibited the cell cycle in the G_2_/M phase.

**Figure 35:**
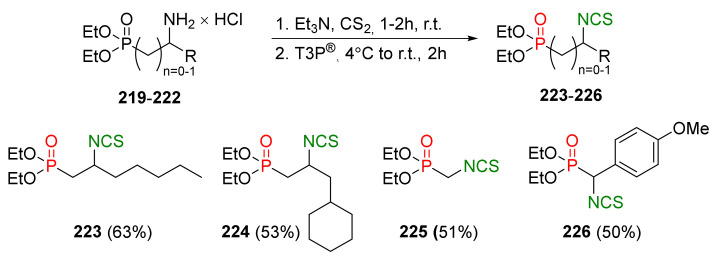
Synthesis of α- and β-dialkoxyphosphoryl isothiocyanates **223**–**226**.

Recently, Janczewski and co-workers [203] developed a one-pot, two-step procedure for the synthesis of phosphorous analogues of SFN **223**–**226** from aminophosphonate hydrochlorides **219**–**222** and carbon disulfide using propane phosphonic acid anhydride (T3P^®^) (**46**) as a desulfurizing agent. Reaction occurred in the presence of triethylamine via the intermediate dithiocarbamates and the target analogues **223**–**226** with the isothiocyanato group in the α- and β-positions, in relation to phosphorus, and with alkyl and aryl substituents obtained in moderate yields (Figure 35).

**Figure 36:**
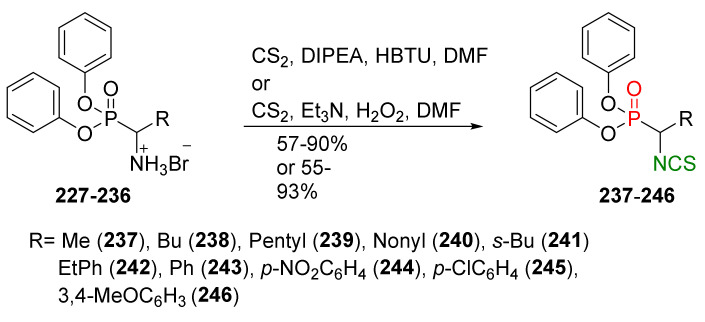
Synthesis of diaryl (1-isothiocyanoalkyl)phosphonates **237**–**246**.

A series of diaryl (1-isothiocyanoalkyl)phosphonates **237**–**246** were prepared and tested for inhibition of human tumor proliferation by Oleksyszyn and co-workers [253]. The starting aminophosphonate hydrobromides **227**–**236** were transformed in an alkaline environment in the presence of CS_2_, and HBTU or H_2_O_2_ as desulfurizing agents into isothiocyanates **237**–**246** in 57–90% yields (with HBTU) or 55–93% (with H_2_O_2_) (Figure 36). Alkyl substituted analogs **237**–**241**, and 3,4-dimethoxyphenyl derivative **246** were obtained in better yields in the reaction with H_2_O_2_, while for the other compounds, **242**–**245** HBTU resulted in slightly higher yields.

The in vitro antiproliferative activities of synthesized ITCs **237**–**246** against LoVo, LoVo/DX, A549, and MCF-7 cell lines were in the range of natural isothiocyanates despite the significant differences in their structures. Among them, ITC **246** was the most active (Table 8).

**Figure 37:**
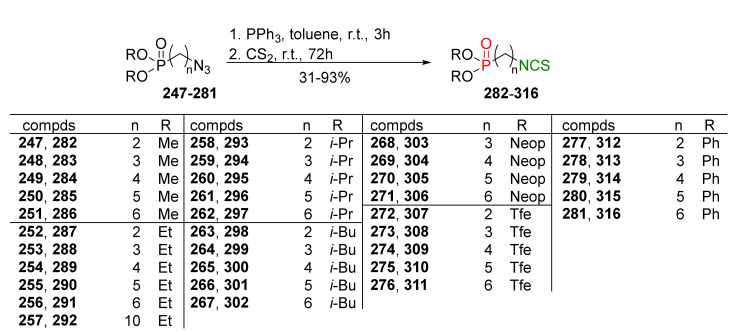
Synthesis of dialkyl and diphenyl ω-(isothiocyanato)alkylphosphonates **282**–**316**.

In 2017, Gajda and Wietrzyk and co-workers [254] designed and synthesized a library of novel bifunctional SFN analogues **282**–**316**, structurally diverse dialkyl, and diphenyl ω-(isothiocyanato)alkylphosphonates (P-ITCs) with an unbranched alkyl side chain containing 2 to 6 carbon atoms. The synthesis of P-ITCs was based on the conversion of dialkyl [255] as well as diphenyl ω-azidoalkylphosphonates **247**–**281** into the target dialkyl and diphenyl ω-(isothiocyanato)alkylphosphonates **282**–**316**, using the tandem Staudinger/aza-Wittig reaction with triphenylphosphine and carbon disulfide with good and very good yields (Figure 37).

**Figure 38:**
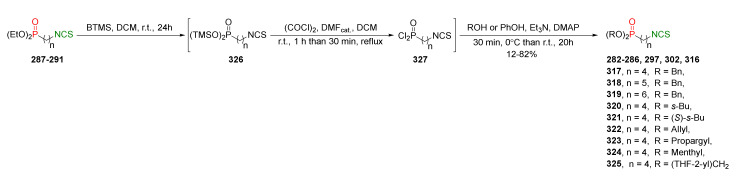
Conversion of diethyl ω-(isothiocyanato)alkylphosphonates **287**–**291** into dialkyl or diphenyl ω-(isothiocyanato)alkylphosphonates **282**–**286**, **297**, **302**, **316**–**325**.

The authors developed a one-pot strategy enabling the direct conversion of ω-(isothiocyanato)alkylphosphonates **287**–**291** into the selected alkyl, or phenyl ω-(isothiocyanato)alkylphosphonates **282**–**286**, **297**, **302**, **316**–**325** in moderate and very high yields (Figure 38).

Dealkylation of diethyl ω-(isothiocyanato)alkylphosphonates **287**–**291** by bromotrimethylsilane (BTMS) generated intermediate bis(trimethylsilyl)alkylphosphonates **326**. Next, crude **326** was converted into the appropriate ω-(isothiocyanato)alkylphosphonic dichlorides **327** treated with oxalyl chloride (COCl)_2_ in the presence of a catalytic amount of DMF. The subsequent reaction of crude dichlorides **327** with the appropriate alcohol or phenol, in the presence of triethylamine, and a catalytic amount of DMAP, provided the target P-ITCs **282**–**286**, **297**, **302**, **316**–**325** in moderate yields (Figure 38).

All synthesized P-ITCs **282**–**325** were evaluated in vitro for antiproliferative activity against the colorectal adenocarcinoma cell line LoVo and its doxorubicin-resistant subline LoVo/DX. SFN, and other natural isothiocyanates, such as BITC or AITC, were used as references. All tested compounds **282**–**325** showed high activity on LoVo and LoVo/DX, higher than natural ITCs (SFN and BITC). The most active were ω-(isothiocyanato)alkylphosphonates with branched isopropyl **293**–**297** and isobutyl **298**–**302** groups on phosphorus. The activity was, for some compounds, more than 10 times higher than SFN activity (Table 9).

In addition, the antiproliferative activity of selected P-ITCs **291**, **297**, and **316** were tested on seven cancer cell lines: murine mammary gland cancer (4T1), leukemia (HL60) and its subline resistant for mitoxantrone (HL60/MX2), non-small lung cancer (A549), uterus sarcoma (MESSA and MESSA/Dx-5), and normal murine fibroblast (BALB/3T3). Compounds **291** (IC_50_ = 0.5 ± 0.2 μM) and **316** (IC_50_= 0.8 ± 0.2 μM) showed similar activity to cisplatin (CDDP) (IC_50_ = 0.4 ± 0.2 μM), relative to HL60 cells. Research with the selected P-ITCs **291**, **297**, and **316**, demonstrated that P-ITCs inhibited the G_2_/M cell cycle on LoVo and LoVo/DX, where P-ITC **316** was more active than SFN, as well as P-ITCs **291**, **297**, and **316**-induced apoptosis. In vivo studies of the selected P-ITCs showed slightly lower anticancer and antimetastatic activity in comparison to naturally occurring BITC. However, recent studies in vivo on Zebrafish have shown that P-SFN **291** was characterized by high anticancer activity and low toxicity [256].

**Figure 39:**
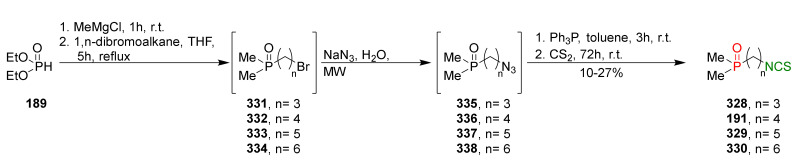
Synthesis of (ω-isothiocyanatoalkyl)dimethylphosphine oxides **191** and **328**–**330**.

The same research group described the synthesis of phosphinates and phosphine oxides analogues of SFN with an unbranched alkyl side chain containing 2 to 6 carbon atoms and with aliphatic and phenyl substituents on the phosphorus atom [257]. The (ω-Isothiocyanatoalkyl)dimethylphosphine oxides **191**, **328**–**330**, were obtained in a three-step reaction following the procedure described by Posner et al. [188]. Thus, crude (ω-bromoalkyl)dimethylphosphine oxides **331**–**334**, prepared in the reaction of diethyl phosphite **189**, with methylmagnesium chloride and 1,n-dibromoalkanes, were subsequently converted into (ω-azidoalkyl)dimethylphosphine oxides **335**–**338** in the microwave-assisted reaction with an aqueous solution of sodium azide. Next, crudes **335**–**338** were converted to (ω-isothiocyanatoalkyl)dimethylphosphine oxides **191**, **328**–**330** in the tandem Staudinger/aza-Wittig reaction with triphenylphosphine and carbon disulfide in low yields (Figure 39).

**Figure 40:**
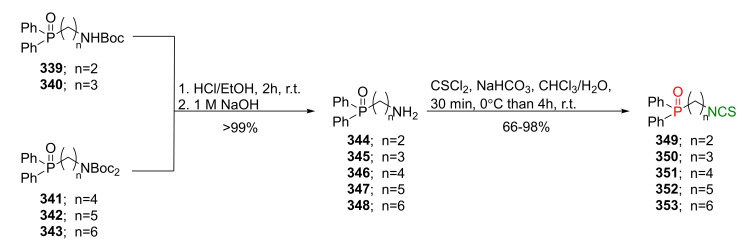
Synthesis of (ω-isothiocyanatoalkyl)diphenylphosphine oxides **349**–**353**.

A different approach was applied for the synthesis of (ω-isothiocyanatoalkyl)diphenylphosphine oxides **349**–**353**. Thus, starting *N*-Boc derivatives **339**–**343** were deprotected under the acidic conditions to afford (ω-aminoalkyl)diphenylphosphine oxides **344**–**348**, quantitatively. The subsequent isothiocyanation of amines **344**–**348** with thiophosgene provided the target (ω-isothiocyanatoalkyl)diphenylphosphine oxides **349**–**353** in good and very good yields (Figure 40).

**Figure 41:**
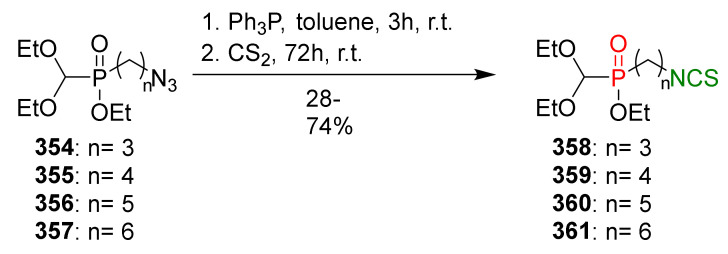
Synthesis of (ω-isothiocyanatoalkyl)(diethoxymethyl)phosphinates **358**–**361**.

**Figure 42:**
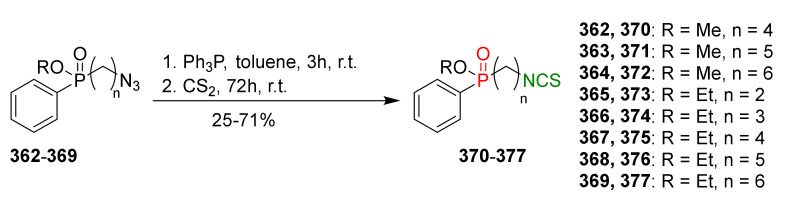
Synthesis of methyl and ethyl (ω-isothiocyanatoalkyl)(phenyl)phosphinates **370**–**377**.

The next group of P-ITCs, such as ethyl (ω-isothiocyanatoalkyl)(diethoxymethyl)phosphinates **358**–**361**, and methyl and ethyl (ω-isothiocyanatoalkyl)(phenyl)phosphinates **370**–**377**, were synthesized from the appropriate (ω-azidoalkyl)phosphinates **354**–**357** and **362**–**369** using the Staudinger/aza-Wittig reaction, with triphenylphosphine and carbon disulfide, in moderate and good yields (Figure 41 and Figure 42).

All P-ITCs **191**, **328**–**330**, **349**–**353**, **358**–**361**, and **370**–**377** were evaluated in vitro for antiproliferative activity against LoVo and LoVo/DX cancer cell lines, and SFN, as well as other natural isothiocyanates, such as BITC or AITC, were used as a reference. Almost all tested compounds, except (ω-isothiocyanatoalkyl)dimethylphosphine oxides **191**, **328**–**330**, showed high activity on LoVo and LoVo/DX, higher than natural ITCs (SFN and BITC). The most active were (ω-isothiocyanatoalkyl)diphenylphosphine oxides **349**–**353** and methyl and ethyl (ω-isothiocyanatoalkyl)(phenyl)phosphinates **370**–**377**, which IC_50_ on LoVo were between 1.8 ± 0.4 and 4.7 ± 1.3 μM and were much more active than SFN and BITC. The antiproliferative activity of the selected P-ITCs **353** and **377** were also tested on 4T1, HL60 and HL60/MX2, A549, MESSA, and MESSA/Dx-5, and BALB/3T3 cell lines, where both P-ITCs showed similar activity. Compounds **353** and **377** were assessed for their mechanisms of action as inducers of the G_2_/M cell cycle arrest and apoptosis. Ethyl (6-isothiocyanatohexyl)(phenyl)phosphinate (**377**) was tested in vivo on the 4T1 cell line and demonstrated moderate antitumor activity, similar to that BITC.

**Figure 43:**
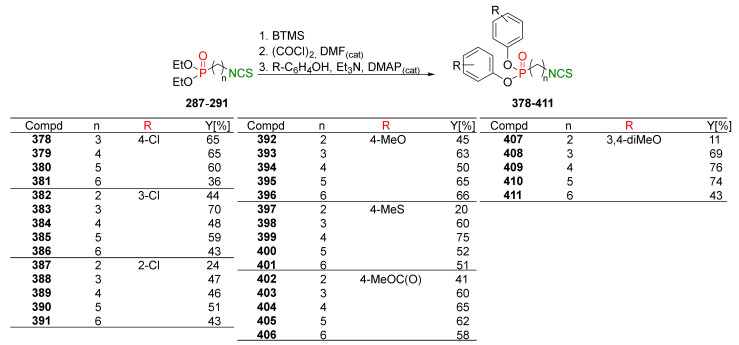
Synthesis of diaryl ω-(isothiocyanato)alkylphosphonates **378**–**411**.

The last paper devoted to the synthesis and biological activity of P-ITCs is from 2019 [258]. The authors synthesized a series of diaryl ω-(isothiocyanato)alkylphosphonates, with a chlorine atom and methoxy, dimethoxy, methylsulfanyl, or methoxycarbonyl groups at *ortho*, *meta*, or *para* positions of the phenyl ring, and with an unbranched alkyl chain **378**–**411** (n = 2–6). Using the same methodology [254] as shown in Figure 38, diethyl ω-(isothiocyanato)alkylphosphonates **287**–**291** were converted in a one-pot reaction with bromotrimethylsilane, oxalyl chloride, and the appropriate phenol derivatives to target diaryl ω-(isothiocyanato)alkylphosphonates **378**–**411** with good yields (Figure 43).

Diaryl ω-(isothiocyanato)alkylphosphonates **378**–**411** were evaluated in vitro for antibacterial activity on *S. aureus* and *P. aeruginosa* strains in comparison with natural PEITC and with gentamicin used as a reference antibiotic. All synthesized P-ITCs characterized high antibacterial activity, higher than natural PEITC. Against the *S. aureus* strain, the most active were **379** (IC_50_ = 1.5 ± 0.1 μM) and **383** (IC_50_ = 2.5 ± 0.2 μM) with activity similar to the gentamicin (IC_50_ = 1.0 ± 0.1 μM). Against the *P. aeruginosa* strain, the most active were **381**, **383**–**384**, **388**–**389**, with IC_50_ between 3.0 ± 2.3 and 4.0 ± 1.6 μM. The selected compounds were three- and four-times more active than gentamicin (IC_50_ = 12 ± 1.0 μM).

Except for antibacterial activity, all P-ITCs were evaluated for antiproliferative activity against LoVo and LoVo/DX cancer call lines. All compounds presented high antiproliferative activity, higher or similar to SFN. The most active was compound **407** (IC_50_= 1.0 ± 0.1 μM), which was 3 times higher than cytostatic CDDP, and more than 22 times higher than SFN.

**Figure 44:**
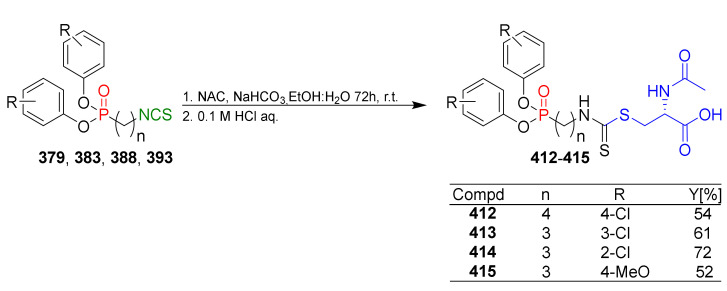
Preparation of phosphonates isothiocyanate-derived mercapturic acids **412**–**415**.

Selected diaryl ω-(isothiocyanato)alkylphosphonates **379**, **383**, **388**, and **393** were also converted into mercapturic acid **412**–**415** derivatives in the reaction with *N*-acetyl-L-cysteine (NAC) and sodium bicarbonate, followed by acidification in good yields (Figure 44).

Preliminary evaluation of the in vitro antibacterial activity on the *S. aureus* strain showed that mercapturic acid **412**–**415** derivatives exhibited moderate antibacterial activity, lower or similar to the parent isothiocyanates **379**, **383**, **388**, and **393**. More accurate research concerning synthesis and antiproliferative activity of phosphonates, phosphinates, and phosphine oxide isothiocyanate-derived mercapturic acids, were described by Psurski and co-workers [259].

### 4.3. Synthesis of Carbonyl and Amide Analogues of Sulforaphane and Their Properties

**Figure 45:**
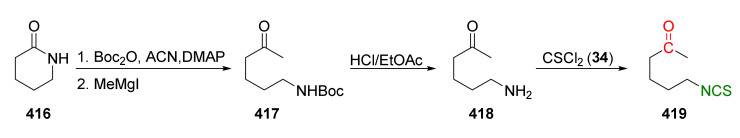
Synthesis of 2-oxohexyl isothiocyanate (**419**).

The next group of SFN analogues are molecules containing carbonyl, ester, and amide moieties. Posner et al. [188] used the same methodology with thiophosgene, shown in Figure 33 for synthesis of carbonyl analogue of SFN, where methylsulfinyl moiety was replaced by carbonyl group (**419**). In this way, δ-valerolactam (**416**), in the reaction with di-*tert*-butyl dicarbonate and methylmagnesium iodide. was transformed into 4-*N*-Boc-aminobutyl methyl ketone (**417**). Ketone **417** under hydrolytic conditions was converted to the 4-aminobutyl-methyl ketone (**418**), followed by the reaction with thiophosgene to afford the 2-oxohexyl isothiocyanate (**419**) with an overall yield of 6% from lactam 413 (Figure 45).

Carbonyl analogue **419** of SFN, as well as SFN (**24**), were evaluated in vitro as inducer potencies of NAD(P)H:quinine oxidoreductase (QR) in murine hepatoma cells (Hepa 1c1c7) (Table 10).

As seen in Table 10, the 2-oxohexyl isothiocyanate (**419**) and SFN (**24**) were the same in potency as the inducer of NAD(P)H:quinine oxidoreductase.

**Figure 46:**
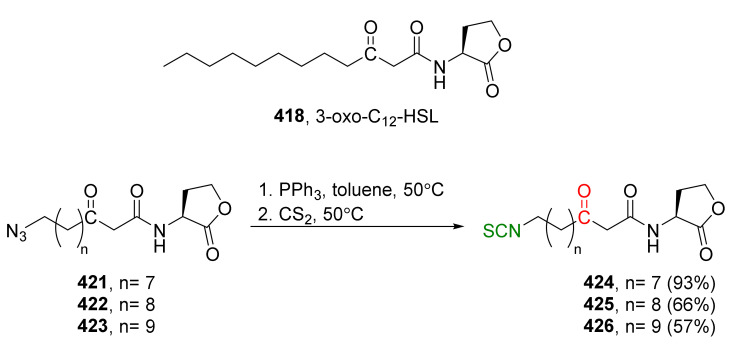
Synthesis of ITCs **424**–**426**.

In 2009, Amara et al. [260] synthesized carbonyl analogues of SFN with a longer alkyl chain containing 8 to 10 carbon atoms, being inhibitors of quorum sensing in *P. aeruginosa.* As a template, the authors used a structure of a natural autoinducer of *P. aeruginosa*–3-oxo-C12-*N*-acyl homoserine lactone (3-oxo-C12-HSL, **420**). Final ITCs **424**–**427** were obtained with good and very good yields from the appropriate azides **421**–**423** in the tandem Staudinger/aza-Wittig reaction with triphenylphosphine and carbon disulfide (Figure 46).

The inhibitions of quorum sensing of *P. aeruginosa* by ITCs **424**–**426** was evaluated using the luminescent PAO1-*luxABCDE* wild type strain. All synthesized ITCs **424**–**426** strongly inhibited luminescence in this wild type strain. The strongest inhibitor of luminescence appeared to be **426** (IC_50_ = 45.2 ± 0.7 μM), followed by **425** (IC_50_ = 113 ± 19 μM) and **424** (~300 μM). Additionally, in the assay with the wild type PAO1 strain, ITC **425** presented significant inhibition of the quorum sensing-controlled virulence factor expression, as well as biofilm formation. The authors showed that the obtained isothiocyanate-based probes covalently and selectively bound Cys79, found in the LasR-binding pocket.

**Figure 47:**
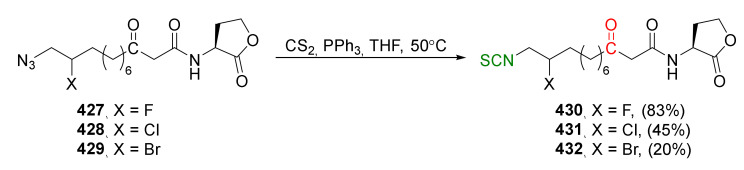
Synthesis of β-halo-ITCs **430**–**432**.

The same research group synthesized the second generation of ITCs based on ITC **425** with halogen moieties (fluorine, bromine, and chlorine) at the β-position [261]. As in previous studies, azides **427**–**429** were transformed into ITCs **430**–**432** in the reaction with triphenylphosphine and carbon disulfide with good yields (Figure 47).

The bioactivity of new ITCs **430**–**432** were evaluated using *P. aeruginosa* PAO1-UW and PAO-JP2 (*lasl/rhll* double mutant) strains carrying the *luxCDABE* cassette as reporters for LasR activation. In this tests, ITC **430** showed more complete inhibition of LasR than parental ITC **425**. On the other hand, ITCs **431** and **432** had higher inhibition of LasR than fluorine-analogue of ITC. Additionally, ITC **430** inhibited pyocyanin production by almost 40%, and its inhibition was stronger than in the presence of ITC **425**. Moreover, ITC **430** caused a swarming inhibition of 44% on agar plates, a stronger effect than that observed for ITC **425**. Other ITCs, **431** and **432**, did not inhibit swarming at all. The authors also showed that, in vivo, ITC **430** increased survival of *C. elegans* from bacterial infections over the course of four days.

**Figure 48:**
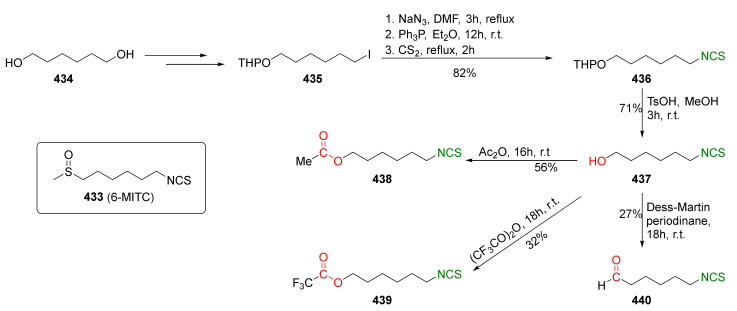
Synthesis of analogues of 6-MITC: ITCs **436**–**440**.

The Staudinger/aza-Wittig reaction was also applied to the synthesis of analogues of 6-(methylsulfinyl)hexyl isothiocyanate (6-MITC, **433**) with the methyl sulfinyl group replaced by another functional group [262]. For this purpose, 1,6-hexanediol (**434**) was transformed in a multi-stage reaction into THP-protected iodide **435**. Then iodide **435**, after prior azidation with NaN_3_, was turned into a THP derivative of ITC **436** with triphenylphosphine and carbon disulfide in an 82% yield. After, deprotection of the tetrahydropyranyl (THP) group with TsOH gave 6-isothiocyanatohexan-1-ol (**437**) in a 71% yield. ITC **437** was used as the starting material in the divergent synthesis of 6-isothiocyanatohexyl acetate (**438**), 6-isothiocyanatohexyl 2,2,2-trifluoroacetate (**439**), and 6-isothiocyanatohexanal (**440**) (Figure 48).

**Figure 49:**
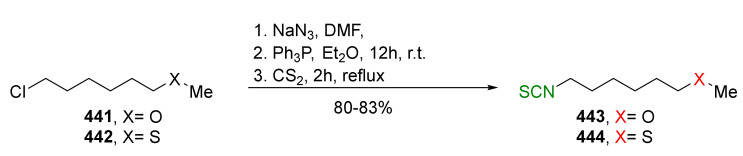
Synthesis of 1-isothiocyanato-6-methoxyhexane (**443**) and 1-isothiocyanato-6-(methylthio)hexane (**444**).

The same sequence of reactions: azidation and reaction with triphenylphosphine/carbon disulfide system, was used for the efficient synthesis of 1-isothiocyanato-6-methoxyhexane (**443**) and 1-isothiocyanato-6-(methylthio)hexane (**444**) from 1-chloro-6-methoxyhexane (**441**) and (6-chlorohexyl)(methyl)sulfane (**442**) (Figure 49).

All synthesized ITCs **437**–**440** and **443**–**444**, as well as thiocyanate **445**, were screened for their antiproliferative and anti-NO production activities, in vitro, using mouse macrophage-like cell line J774.1 cells (Table 11).

Compounds **437**–**439** and **443** had almost similar antiproliferative and anti-NO production activities at those of ITC **433**. ITCs **440** and **444** were less active than ITC **443**. Thiocyanate **445**, compared with ITC **433**, had no activity, which shows that the isothiocyanate group is responsible for biological activity.

**Figure 50:**
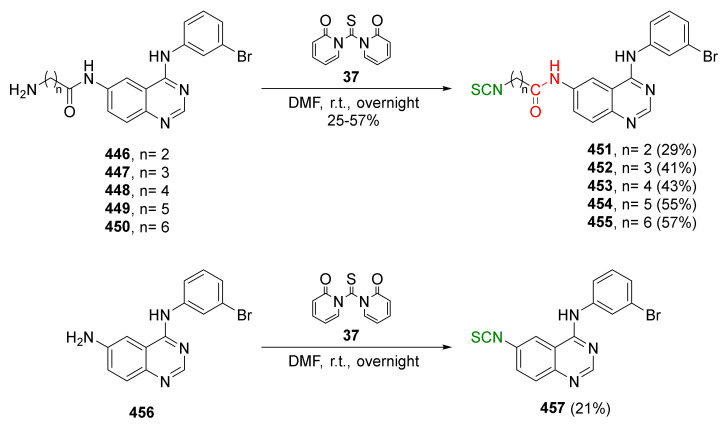
Synthesis of ITCs **451**–**455** and **457**.

Milelli, Minarini, and co-workers [263] described synthesis of novel quinazoline derivatives with polymethylene-amide linkers of different lengths, and possessing an isothiocyanate group, **451**–**455**, or derivatives, such as **457**, where NCS moiety was directly connected to the aromatic ring. In substrates **446**–**450** and **456**, isothiocyanation of amino groups was accomplished with 1,1′-thiocarbonyldi-2,2′-pyridone (**37**) in satisfactory yields (Figure 50).

ITCs **451**–**455** and **457** were evaluated for their ability to inhibit the proliferation of the highly epidermal growth factor receptor (EGFR-TK) expressed in human epithelial cancer cells A431 and HaCaT cancer cells. ITCs **451**–**455** had similar activity on the A431 cell line (IC_50_ between 14.38 ± 1.5 μM and 17.06 ± 1.87 μM) to SFN (IC_50_ = 15.76 ± 1.89 μM), and also similar activity on the HaCaT cell line. The most active was ITC **457**, which IC_50_ on the A431 cell line was much higher than SFN and other ITCs (IC_50_= 2.04 ± 0.22 μM) and also higher on HaCaT (IC_50_ = 2.62 ± 0.31 μM). Which ITC **457** did not affect the normal cell proliferation of human fibroblasts (HGFs) is noteworthy. The inhibitory effect of new ITCs **451**–**455** and **457** on EGFR-TK activity evaluated on A431 cell lysates showed that ITC **457** had inhibition effects at the nanomolar concentrations with a maximum inhibition of 89% at 10 μM. ITCs **451**–**455** were weaker inhibitors. Additionally, treatment of A431 cells with ITC **457** at 2 μM led to apoptosis, in terms of DNA fragmentation.

**Figure 51:**
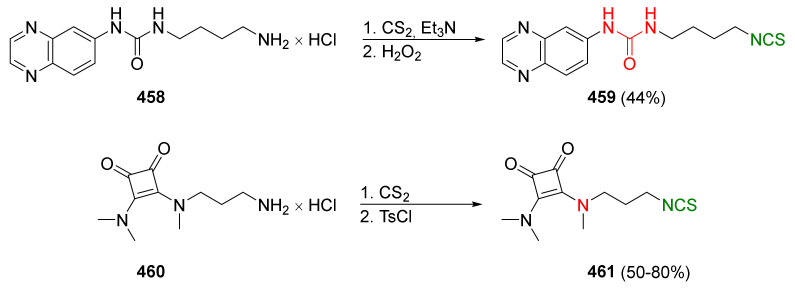
Synthesis of SFN analogues **459** and **461**.

In 2019, Boehm and co-workers [264] synthesized crystalline SFN analogues **459** and **461**. Both isothiocyanates **459** and **461** were obtained in good yields, in a two-step reaction of the appropriate amine hydrochlorides **458** or **460**, and carbon disulfide in the presence of triethylamine, using hydrogen peroxide or tosyl chloride as desulfurizing agents (Figure 51).

The dialkyl tertiary squaramide **461** isolated as a crystalline solid with good solid-state stability was shown to be a covalent Nrf2 activator that binds to the BTB domain of KEAP1. Additionally, ITC **461** has demonstrated efficiency for activation of the Nrf2 pathway in human bronchial epithelial (BEAS2B) cells, translating to a dose-dependent inhibition of lung inflammation in an in vivo model of pulmonary oxidative stress.

**Figure 52:**
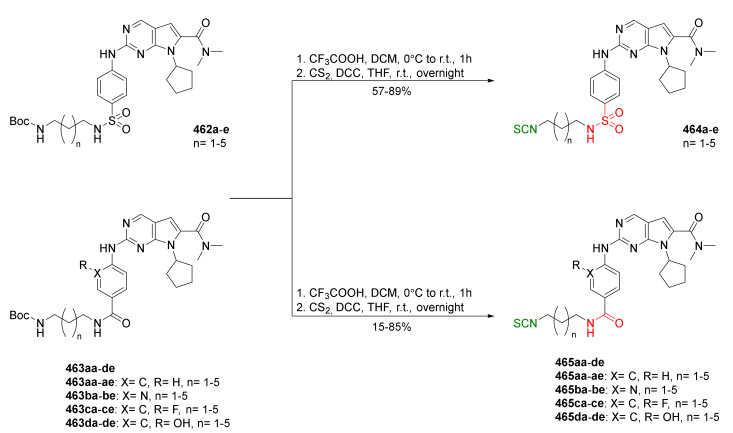
Synthesis of ITCs **464a**–**e** and **465aa**–**de**.

**Figure 53:**
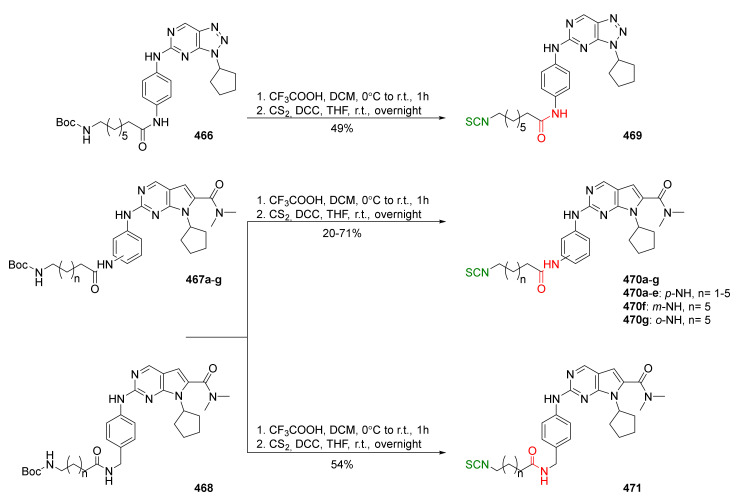
Synthesis of ITCs **469**, **470a**–**g**, and **471**.

Recently, Wang and co-workers [265] designed and synthesized a series of cyclin-dependent kinase 9 (CDK9) inhibitors with cancer stem cell (CSC) inhibition activity for non-small-cell lung cancer (NSCLC) therapy. The structures of the inhibitors were based on the combination of the pyrrolo-[2,3-*d*]pyrimidines-2-amine from ribociclib as CDK9 pharmacophore, and analogues of SFN with alkyl chains of different lengths as targeting CSC pharmacophore. Briefly, the key *N*-Boc amines **462a**–**e** and **463aa**–**de** were converted into the final ITCs **464a**–**e** and **465aa**–**de** after prior deprotection of the amino group by trifluoroacetic acid, followed by the reaction with carbon disulfide and *N,N’-*dicyclohexylcarbodiimide (DCC, **45**) (Figure 52). The same methodology was used to transform amines **466**, **467a**–**g**, and **468** into ITCs **469**, **470a**–**g**, and **471** (Figure 53).

All ITCs **464a**–**e**, **465aa**–**de**, **469**, **470a**–**g**, and **471** were evaluated regarding inhibition activity against CDK4, CDK6, and CDK9, with ribociclib as the positive control. Some compounds showed high potent activity against CDK9 as well as high selectivity for CDK9. The inhibition of tested ITCs was similar or higher than inhibition of ribociclib. In particular, ITC **470e** with an 8-isothiocyanatooctanamide linker, exhibited high enzymatic inhibition (IC_50_ = 11 nM vs. IC_50_ of ribociclib = 197 nM). ITC **470e** was tested against several tumor cell lines, including NSCLC, breast cancer, hepatocarcinoma, cervical cancer, leukemia, and lymphoma, using CCK8 assay and SFN as positive controls. ITC **470e** had the highest activity against NSCLC cell lines, especial A549 and H1299, with IC_50_ values less than 0.5 μM. For comparison, IC_50_ of SFN on A549 was more than 10 μM and IC_50_ of ribociclib was 7.455 μM. For other cell lines, ITC **470e** exhibited lower anti-viability activity. These results demonstrated good cellular selectivity of ITC **470e**. Additionally, studies on A549 and H1299 showed that ITC **470e** dose-dependently inhibited the cell cycle at G_2_/M phase and induced apoptosis of A549 cells in a concentration-dependent manner. Moreover, ITC **470e** decreased the formation of colonies in the two NSCLC cell lines. In vivo studies on H1299 xenograft mouse models showed that ITC **470e** displayed potent anti-tumor activities.

### 4.4. Synthesis of Ether-Linked Analogues of Sulforaphane and Their Properties

**Figure 54:**
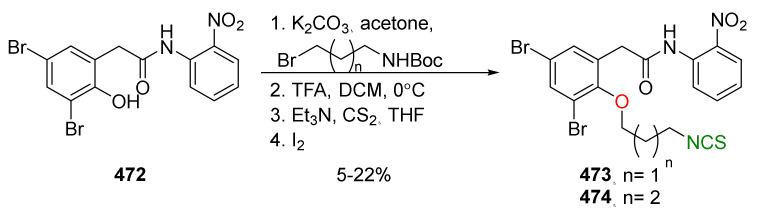
Synthesis of ether-linked isothiocyanate analogues **473** and **474**.

Perez and co-workers [266] synthesized ether-linked isothiocyanate as the LasR antagonist of quorum sensing in *P. aeruginosa.* Briefly, etherification of phenol **472**, followed by amino group deprotection with TFA and a two-step reaction with carbon disulfide and molecular iodine, afforded ether-linked isothiocyanates **473** and **474** in low yields (Figure 54). A LasR antagonist bioassay exhibited low IC_50_ values (ITC **473** IC_50_ > 200 μM and ITC **474** IC_50_ = 145 ± 105 μM) with no effect on bacterial growth.

**Figure 55:**
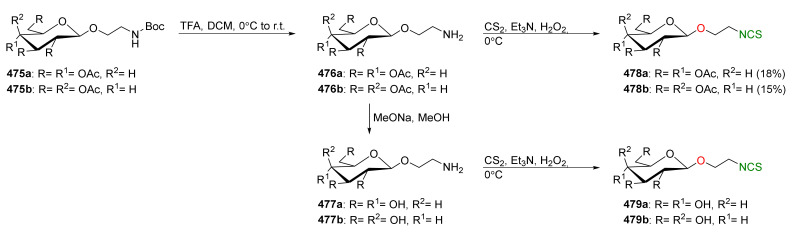
Synthesis of the target ITCs derivatives **478a**–**b** and **479a**–**b**.

In 2019, Bussolo and Minutolo et al. [267] synthesized novel glycoconjugated H_2_S donors with the isothiocyanate group. For this purpose, the starting *N*-Boc-glycoconjugates **475a**–**b** were deprotected with TFA, followed by the reaction of amino derivatives **476a**–**b** with an excess of carbon disulfide, triethylamine, and hydrogen peroxide to afford final, fully acetylated ITCs **478a**–**b**, with low yields. On the other hand, deacetylation of *O*-acetylated amines **476a**–**b** with sodium methanolate, followed by the isothiocyanation of amines **477a**–**b**, performed in the same manner as above, gave isothiocyanate glycopyranosides **479a**–**b** in 21% and 22% yields, respectively (Figure 55).

ITCs **478b** and **479a** were evaluated for their cell inhibitory effects on viability, using pancreatic adenocarcinoma tumor cells (AsPC-1). Studies showed that glucose-derivative **479a** was totally ineffective at inhibiting cell viability. However acetyl-galactosamine-derivative **478b** exhibited a marked cytotoxicity against AsPC-1 tumor cells in a concentration-dependent manner with a pIC_50_ value of 4.45 ± 0.02. ITC **478b** efficiently released H_2_S intracellularly with desirable slow kinetics and inhibition of cell cycle progression G_0_/G_1_ phase.

**Figure 56:**
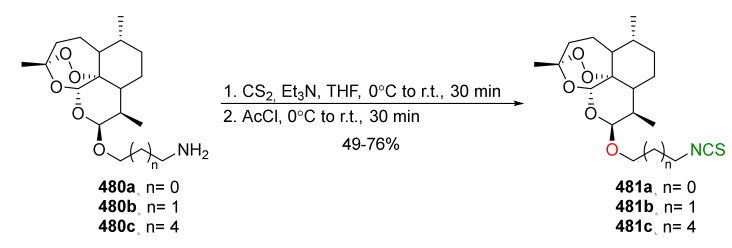
Synthesis of artemisinin–isothiocyanate derivatives **481a**–**c**.

He and co-workers [268] designed and synthesized artemisinin–isothiocyanate derivatives with anti-glioblastoma effects. The artemisinin–isothiocyanate derivatives **481a**–**c** were obtained in good yields from amines **480a**–**c** in a two-step reaction with carbon disulfide and triethylamine used as a base, followed by desulfurization with acyl chloride (Figure 56).

All ITCs **481a**–**c** showed higher anti-tumor effects in vitro than dihydroartemisinin (DHA) against human glioblastoma U87 cells. The most active was ITC **481b** (IC_50_ = 7.41 ± 1.56 μM) (IC_50 DHA_ = 118.95 ± 12.39 μM). ITC **481b** reduced the viability of glioblastoma multiforme (GBM) in a concentration-dependent manner. ITC **481b** also inhibited migration and induced apoptosis in U87 cells. Pyknosis and nuclear shrinkages were observed in ITC **481b**-treated cells. Additionally, caspase 9 and cytochrome *c* were induced in U87 cells treated with ITC **481b** and cleaved caspase 3 was increased. On the other hand, the anti-apoptotic protein Bcl-2 was downregulated, and pro-apoptotic protein BAX was upregulated.

### 4.5. Synthesis of Diisothiocyanates

**Figure 57:**
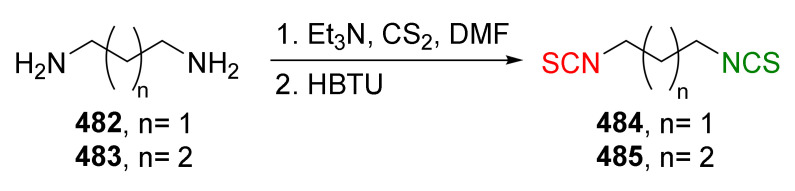
Synthesis of diisothiocyanates **484** and **485**.

Oleksyszyn and co-workers [269] synthesized a library of diisothiocyanates (diITCs) as well as their mercapturic acid derivatives conjugated with *N*-acetyl cysteine. Both isothiocyanate functional groups in diITCs were separated with unbranched alkyl linkers containing three and four carbon atoms (**484** and **485**). DiITCs **484** and **4825** were obtained from the corresponding diamines **482** and **483** in a two-step procedure with carbon disulfide and triethylamine as a base, followed by the reaction with HBTU as desulfurizing reagents (Figure 57).

Synthesized diITC were evaluated in vitro on LoVo and LoVo/DX cancer cell lines. Their IC_50_ values on the LoVo cancer cell line were very high and were lower than 2 μM. Additionally, their biological activities were higher than their mercapturic acid derivatives.

**Figure 58:**
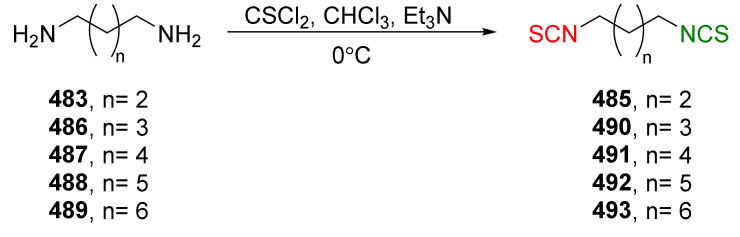
Synthesis of diisothiocyanates **485**, **490**–**493**.

Diisothiocyanates were also obtained by Mustaev and co-workers [55]. The authors synthesized diITC **485**, **490**–**493**, with unbranched alkyl linker containing four to eight carbon atoms from the corresponding diamines **483**, **486**–**489**, and thiophosgene in the presence of triethylamine as a base (Figure 58).

The growth-inhibitory activity of synthesized diITCs **485**, **490**–**493**, against pathogenic bacteria, fungi, and molds were evaluated. DiITCs **485**, **490**–**493**, were more active against Gram-positive bacteria (*B. cereus*; MIC = 2 μg/mL) than Gram-negative (*E. coli*; MIC > 64 μg/mL). Studies on wide selections of Gram-negative as well as Gram-positive strains showed that the most active were diITCs **492** and **493**, which, on Gram-positive strains, such as *Streptococcus*, *Staphylococcus,* and *Bacillus,* exhibited high activity (MIC = 1–2 μg/mL). DiITCs **492** and **493** presented high activity against mycobacteria. The most active were against *M. bovis* and *M. tuberculosis* (MIC = 2–4 μg/mL) than against nontuberculous mycobacteria. DiITC **492** efficiently inhibited the growth of the pathogens, such as *Candida albicans* or *Candida glabrata,* for which MIC < 1 μg/mL.

### 4.6. Summary of the Synthetic Routs of SFN and Its Bifunctional Analogs and Their Biological Activity 

The described above methods of the synthesis of SFN and its bifunctional analogs are presented in Table 12, divided into various functional groups.

Based on the Table 12, it can be seen that the synthesis of SFN, and its sulfur analogs substituted with the methyl group, but also by varied aliphatic, aromatic, fluorine, or heterocyclic substituents, are the most described in the literature. Among them, the method using thiophosgene (CSCl_2_) is dominant. The next often used method is the tandem Staudinger/aza-Wittig reaction, and the least frequently used is the two-step method using a desulfurizing agent. Another group involves phosphorus analogues of SFN (P-SFN) with phosphonate, phosphinate, or phosphine oxide moiety with aliphatic or aromatic substituents. For this class of compounds, both the tandem Staudinger/aza-Wittig reaction and the method using a desulfurizing agent were equally frequently used. The reaction with CSCl_2_ for synthesis P-SFN was used less frequently. Carbonyl and amide analogs of SFN were synthesized by all three methods. The synthesis of ether-linked analogs of SFN was completely performed by the two-step methodology with a desulfurizing agent, while the synthesis of diisothiocyanates was also performed with the desulfurizing agent and thiophosgene. The tandem Staudinger/aza-Wittig reaction, to the synthesis of ether-linked analogs of SFN and diisothiocyanates, was not used.

Table 13, summarizes the relationship between the biological activity of analogs of SFN and various functional groups. The table presents the most anticancer-active analogs of SFN containing sulfinyl, phosphonate, phosphinate, phosphine oxide, carbonyl, carboxamide, or additional isothiocyanate functional groups. Due to the fact that there are no differences in biological activity between SFN and its natural analogs, such as erucin or alyssin (see Figure 5), these compounds are not placed in Table 13. Only the analogs of SFN are placed in Table 13 for which biological activity is compared with SFN.

Based on Table 13, it can be seen that replacing the methyl group in sulfinyl moiety in SFN with structurally diverse fluorine ((S)-**75**, **80d**), heterocyclic (**84d**), or aliphatic (e.g., iso-butyl) (**152**) substituents result in increases of the biological activities of analogs of SFN on different cancer cell lines, such as Malme-3M, MCF-7, or A549. For phosphorus analogs of SFN (P-SFN), the presence of phosphine oxide moiety with methyl substituents (**191**) does not affect biological activity. However, replacement of the methyl sulfinyl group by phosphonate moiety with aliphatic (**298**) or aromatic (**407**) substituents or phosphinate moiety (**372**) results in an increase in the anticancer activity on LoVo and LoVo/DX cancer cell lines. Carbonyl or amide analogs of SFN (**419** and **455**) can by characterized by biological activities similar to SFN, but on the other hand, for some analogs (e.g., **470e**), biological activity could be higher than for SFN. The presence of the second isothiocyanato group (**484**) also results in an increase in the biological activity on LoVo and LoVo/DX cancer cell lines.

## 5. Conclusions

There are many examples describing the synthesis of SFN and its natural or synthetic difunctional analogues, with methyl sulfinyl group replaced by sulfinyl, sulfanyl, sulfonyl, phosphonate, phosphinate, phosphine oxide, carbonyl, ester, amide, ether, or a second isothiocyanate group. The most common strategies involve the use of thiophosgene as a thiocarbonyl transfer reagent, utilization of a carbon disulfide/desulfurizing agent system (dithiocarbamates approach), or use of the tandem Staudinger/aza-Wittig reaction (triphenylphosphine/carbon disulfide system). In the first two approaches, primary amines are utilized as substrates; the last comprises azides as starting materials. The choice of method depends on the availability of starting substrates and sensitivity of functional groups present in the substrates to the reaction conditions. Several ITCs are characterized by high biological activity (anticancer or antibacterial), usually higher than parental SFN. Which modifications of the structure of SFN seem to have important impacts on the biological activities of the new analogues of SFN.

As SFN is biologically active, natural ITC is found in cruciferous vegetables, and it is non-toxic—it has been selected for phase I and II clinical trials, where it is administered in the form of an extract or broccoli sprouts. The results of these studies are promising and indicate which SFN, in the future, may be considered as an anticancer drug. The situation is different for synthetic analogs of SFN, which have been described in this review, having sulfinyl, sulfanyl, sulfonyl, phosphonate, phosphinate, phosphine oxide, carbonyl, ester, amide, ether, or a second isothiocyanate group. The vast majority of these compounds have only been tested for anticancer or antibacterial activity in vitro, and a few analogs, additionally in vivo, on mice or Zebrafish. None of the synthetic analogs of SFN described in this review qualified for clinical trials. This is likely due to the toxicity of these compounds in higher doses, often seen in in vitro or in vivo studies. SFN, which is a natural product, does not show toxicity, even in higher doses. Another reason is the financial constraints of many research teams.

One interesting solution would be to synthesize a conjugate—a combination of SFN and a drug or peptide, with anticancer properties, characterized by no (or low) toxicity. This conjugate could separate, in cancer cells, on two drugs, causing increased anticancer activity.

## Figures and Tables

**Table 1:** Cytotoxicity of SFN (**24**), erucin (**28**), and erysolin (**31**).

Compound	Low IC_50_ (μM)	High IC_50_ (μM)	IC_50_ (NmuMG) (μM)
SFN **(24)**	2.8 ± 0.1 (Hep3B)	16.5 ± 1.4 (NCI/ADR RES)	3.2 ± 0.2
Erucin **(28)**	8.9 ± 0.4 (SF-268)	45.3 ± 4.9 (HCI-H460)	23.5
Erysolin **(31)**	2.3 ± 0.8 (MCF-7)	11.1 ± 0.6 (NCI/ADR RES)	5.3 ± 0.4

**Table 2:** Cytotoxicity of SFN and its fluorine-containing analogs **75**.

Compound	Malme-3M IC_50_ (μM)	Malme-3 IC_50_ (μM)
(R)-SFN	25	38
(*S*)-SFN	30	26
(*R*)-75	27	37
(*S*)-75	25	35

**Table 3:** The IC_50_ (μM) for **80d** and **80e** and SFN after 72 h of incubation (mean ± SD).

	IC_50_ (μM) ± SD			
**Compound**	**MALME-3M**	**HT-29**	**MCF-7**	**MDA-MB-231**
**80d**	2.7 ± 0.7	1.2 ± 0.1	0.9 ± 0.1	0.5 ± 0.1
**80e**	4.3 ± 0.7	1.4 ± 0.2	0.7 ± 0.1	1.2 ± 0.1
SFN	33.7 ± 0.6	11.4 ±0.1	11.9 ± 2.0	11.3 ± 0.7

**Table 4:** Inhibitory effects of tetrazole analogues of SFN **83d**, **84d**, and **89d** and SFN on MCF-7, SUM-159, and KG-1a.

Compound	MCF-7 IC_50_ (μM)	SUM-159 IC_50_ (μM)	KG-1a IC_50_ (μM)
SFN	24.11 ± 6.62	7.69 ± 0.92	8.24 ± 2.81
**83d**	2.66 ± 0.25	1.46 ± 0.19	1.52 ± 0.38
**84d**	4.11 ± 0.9	1.54 ± 0.29	0.51 ± 0.14
**89d**	1.66 ± 0.23	2.08 ± 0.24	0.88 ± 0.28

**Table 5:** The cytotoxicity of SFN and its derivatives.

Compound	HepG2 IC_50_ (μM)	A549 IC_50_ (μM)	MCF-7 IC_50_ (μM)	HCT-116 IC_50_ (μM)	SH-SY5Y IC_50_ (μM)
**SFN (24)**	14.05	21.99	17.66	11.59	13.72
	2.05 **(151)**	5.64 **(161)**	3.3 **(155)**	2.06 **(152)**	2.79 **(157)**
**135**	12.56	51.34	38.28	41.45	20.71
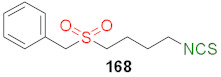	8.49	8.89	7.55	6.28	4.78

**Table 6:** Selectively of (*S*)-SFN and (*S*)-**184**.

Compound	A549 IC_50_ (μM)	MRC-5 IC_50_ (μM)
(*S*)-SFN ((*S*)-**24**)	19.60	46.58
(*S*)-**184**	7.54	17.58

**Table 7:** Effects of SFN and its analogues **191** on inducer potency for QR in Hepa 1c1c7.

Compound	CD (μM)
SFN **(24)**	0.2
**191**	0.4

**Table 8:** The antiproliferative activity of compound **246**.

IC_50_ (μM) ± SD			
LoVo	LoVo/DX	A549	MCF-7
7 ± 1	8 ± 1	31 ± 2	20 ± 1

**Table 9:** Antiproliferative activity of isopropyl and isobutyl ω-(isothiocyanato)alkylphosphonates **293**–**302**.

Compound	LoVo IC_50_ (μM)	LoVo/DX IC_50_ (μM)	Compound	LoVo IC_50_ (μM)	LoVo/DX IC_50_ (μM)
**293**	2.7 ± 0.4	3.6 ± 0.9	**298**	1.9 ± 0.4	2.6 ± 0.4
**294**	2.6 ± 0.2	3.3 ± 0.9	**299**	2.4 ± 0.3	5.6 ± 3.2
**295**	2.5 ± 0.6	5.0 ± 3.7	**300**	3.3 ± 0.2	10.4 ± 1.4
**296**	2.6 ± 0.1	7.9 ± 1.6	**301**	2.4 ± 0.5	5.1 ± 4.4
**297**	2.7 ± 0.2	9.4 ± 1.6	**302**	2.7 ± 0.4	9.2 ± 1.4
			**SFN**	22.9 ± 2.0	18.1 ± 3.0

**Table 10:** Effect of SFN and its analogue **419** on the inducer potency for QR in Hepa 1c1c7.

Compound	CD (μM)
SFN **(24)**	0.2
**419**	0.2

**Table 11:** In vitro NO production and tumor growth inhibitory activities of the most active ITCs **433**, **437**–**439**, **443**, and thiocyanate **445**.

Compounds	Inhibition (IC_50_, μM)	
	NO Production	Growth
433 (6-MITC)	5.7 ± 0.5	8.0 ± 0.6
437	6.0 ± 1.2	4.4 ± 0.3
438	6.6 ± 1.2	4.1 ± 0.2
439	9.1 ± 1.0	5.6 ± 0.4
443	11.5 ± 5.9	8.1 ± 0.6
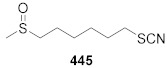	> 200	> 200

**Table 12:** Summary of the synthetic routes of SFN and its bifunctional analogs.

*Reaction*	Reference
*Synthesis of SFN and its sulfur analogues*	
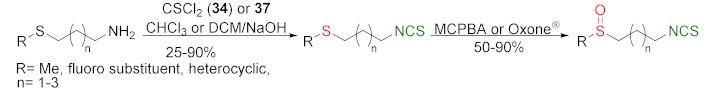	[239,240,241,242,243,244,245,246,247]
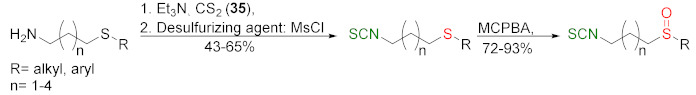	[249]
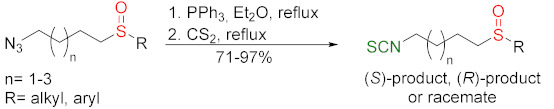	[163,250,252]
*Synthesis of phosphorous analogues of SFN*	
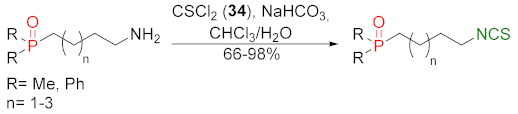	[188,257]
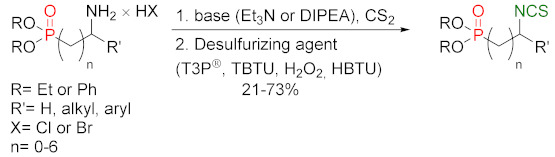	[200,203,253]
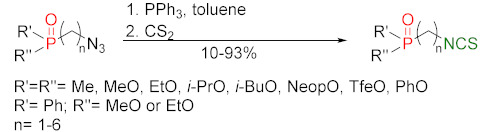	[200,254,257]
*Synthesis of carbonyl and amide analogues of SFN*	
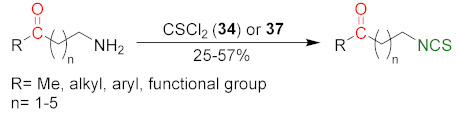	[188,263]
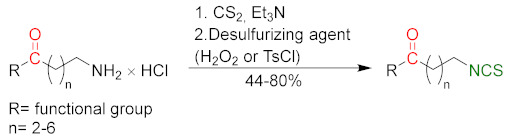	[264,265]
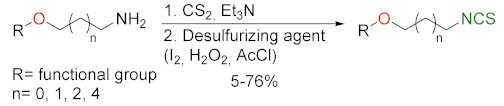	[260,261,262]
*Synthesis of ether-linked analogues of SFN*	
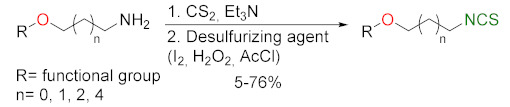	[266,267,268]
*Synthesis of diisothiocyanates*	
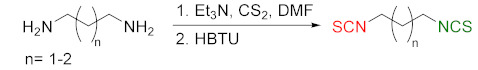	[269]
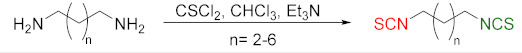	[55]

**Table 13:** Summary of the relationship between the biological activities of the analogs of SFN and various functional groups.

Compound	Anticancer Activity	Reference	Compound	Anticancer Activity	Reference
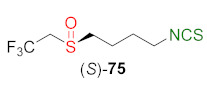	Malme-3M↑ Malme-3↓	[244]	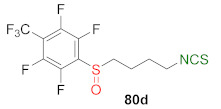	Malme-3M↑ HT-29↑ MCF-7↑ MDA-MB-231↑	[245]
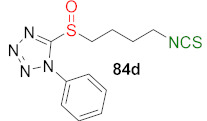	MCF-7↑ SUM-159↑ KG-1a↑	[246]	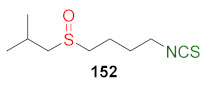	HepG2↑ A549↑ MCF-7↑ HCT-116↑ SH-SY5Y↑	[249]
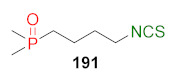	CD↔	[188]	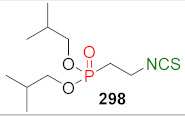	LoVo↑ LoVo/DX↑	[254]
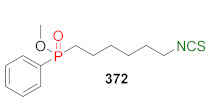	LoVo↑ LoVo/DX↑	[257]	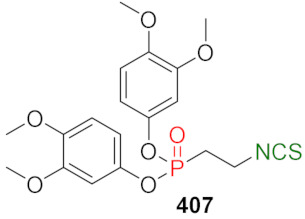	LoVo↑ LoVo/DX↑	[258]
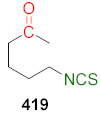	CD↔	[188]	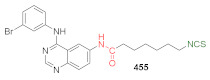	A431↔	[263]
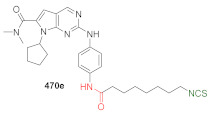	A549↑ H1299↑ MCF-7↑ MDA-MB-231↑ HepG2↑ Hela↑	[264]	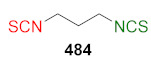	LoVo↑ LoVo/DX↑	[269]

↑—more active than SFN; ↓—less active than SFN; ↔—similar active to SFN.

## Data Availability

The data presented in this study are available upon request from the corresponding author.
